# Does intervening to improve early academic adversity impact later mental health during adolescence? A population-based linked data modelling study in Wales

**DOI:** 10.1136/bmjopen-2026-119243

**Published:** 2026-08-14

**Authors:** Sebastian Stannard, Ann Berrington, Nida Ziauddeen, Simon DS Fraser, Nisreen A Alwan

**Affiliations:** 1 School of Primary Care, Population Sciences and Medical Education, Faculty of Medicine, University of Southampton, Southampton, UK; 2 NIHR Applied Research Collaboration Wessex, Southampton, UK; 3 School of Economic, Social and Political Sciences, University of Southampton, Southampton, UK; 4 NIHR Southampton Biomedical Research Centre and University Hospital Southampton NHS Foundation Trust, University of Southampton, Southampton, UK; 5 University Hospital Southampton NHS Foundation Trust, Southampton, UK

**Keywords:** Adolescent, MENTAL HEALTH, PUBLIC HEALTH, EPIDEMIOLOGY

## Abstract

**Abstract:**

**Objective:**

The UK is facing a child and adolescent mental health crisis. We explored academic adversity recorded in year 6 in relation to diagnosed anxiety or depression in adolescence (ages 13–18). We then modelled the effect of real-world interventions aimed at addressing academic adversity, including Sure Start and universal free school meals combined, on mental health outcomes.

**Methods and analysis:**

Our sample consisted of children in the Welsh Secure Anonymised Information Linkage (SAIL) databank that includes individuals born between 1 January 2000 and 31 December 2022. The outcome was a first diagnosis of anxiety or depression between ages 13 and 18 identified through primary care records or hospital admissions. We constructed an adversity score within the school and academic ability domain including attainment, attendance, exclusion and teacher–pupil ratio and used adjusted multivariable logistic regression to examine the relationship with the outcomes. We generated adjusted population attributable fractions (PAFs) to estimate the reduction in outcome risk if adversity scores were reduced. Using combined effect estimates from evaluations of Sure Start and universal free school meals, we calculated the absolute reduction in the outcome risk had children been exposed to both interventions.

**Results:**

Among adolescents aged 13–18, 2.8% (6620/233 329) of boys and 7.2% (16 034/221 622) of girls had a first diagnosis of anxiety, while 2.7% (6463/236 165) of boys and 6.4% (14 276/224 462) of girls were diagnosed with depression. In fully adjusted models, higher academic adversity scores (across all levels) in year 6 were significantly associated with increased odds of anxiety for both boys and girls. For depression, higher adversity (across all levels) was associated with increased odds among girls, while highest adversity (2+) was associated with depression among boys. PAFs suggested that reducing adversity could lead to modest reductions in the reporting of anxiety and depression, with greater potential impact for boys. Hypothetical exposure to combined early-life interventions (Sure Start and free school meals) resulted in small absolute reductions in anxiety diagnoses (0.6% (11/1816)–1.6% (12/787) for boys; 0.3% (12/3986)–0.5% (11/1080) for girls) and a 0.4% (16/3636) reduction in the reporting of depression diagnoses for girls, with no measurable impact for boys.

**Conclusion:**

Interventions providing support during childhood may have a modest impact on reducing anxiety in adolescence for boys, and both anxiety and depression for girls.

**STRENGTHS AND LIMITATIONS OF THIS STUDY**

- This study aimed to use a stepped-based approach to model the impact of childhood academic adversity and related real-world interventions on adolescent mental health using a large, population-level linked cohort from the Secure Anonymised Information Linkage databank.
- A domain-based approach to modelling academic adversity represents a novel method that captures interconnected school-level and individual factors.
- The use of multivariable logistic regression with extensive adjustment for perinatal, socioeconomic, health and school-level covariates strengthens internal validity, although residual confounding cannot be excluded.
- Intervention effects were modelled using published estimates from Sure Start and universal free school meals, providing a pragmatic approach but relying on assumptions about transferability and combined effects.
- The analysis was restricted to routinely collected data and a Welsh population, which may limit generalisability and is subject to missing data and selection bias.

## Background

 More than one in five children and young people in England now have a diagnosable mental health condition,^1^ and the number of children requiring support from Children and Adolescent Mental Health Servies in England has increased by nearly 53% since 2018.^2^ In addition to putting a strain on the National Health Service (NHS) and young persons’ services,^3^ poor childhood mental health is linked to worse outcomes across the life course including substance misuse, physical health problems, loneliness, lower life satisfaction, unemployment and increased healthcare utilisation.^4–6^

A range of early life factors are known to shape mental health outcomes in adolescence. These include individual characteristics such as self-esteem and self-efficacy,^7^ as well as family-related factors, including parental mental health conditions and genetic predispositions.^8 9^ Adverse childhood experiences (ACEs) and aspects of the family environment such as socioeconomic status, parental divorce or separation, abuse and neglect, domestic violence and family dynamics also influence adolescent mental health outcomes.^10 11^ Across the life course broader structural conditions and social determinants such as income, employment, food security, housing, social support, discrimination and neighbourhood environments are important influences on mental health.^12^ However, the contribution of early academic and educational indicators, such as primary school attendance and attainment, on subsequent mental health outcomes in adolescence is not well understood.

A co-produced workshop involving 25 stakeholders with expertise in early life policy and practice,^13^ highlighted the increasing pressure that childhood mental health issues are placing on secondary schools and child services. Stakeholders identified key transitional periods, such as the end of primary school, as practical opportunities to identify children at risk of poor mental health outcomes. These transitional periods were seen as critical windows for the timely implementation of preventive interventions. In addition, school attendance and academic attainment were emphasised as pivotal factors influencing childhood health—a view supported within the literature.^14 15^ Stakeholders discussed the importance of school as a universal touchpoint for intervention, given the strong correlation between poor attendance, low attainment and adverse health outcomes.^13^

Building on this stakeholder workshop, the aim of this paper was to explore whether a domain incorporating factors relating to academic adversity recorded in year 6 (ages 10–11) is associated with diagnosed anxiety or depression in adolescence (ages 13–18). We then modelled prevention scenarios within this domain on the outcome of anxiety or depression using effectiveness data of real-world interventions addressing academic adversity. These interventions included free school meals (FSM) and Sure Start (later known as Flying Start in Wales), an early years programme introduced in 1998 in areas based on socioeconomic deprivation to provide integrated support to families with children under five, including health services, parenting support, early learning and childcare.

## Methods

This paper was an analysis of longitudinal children’s data using linked records between 2000 and 2022. It was conducted as part of a research collaboration titled Multidisciplinary Ecosystem to study Lifecourse Determinants and Prevention of Early-onset Burdensome Multimorbidity (MELD-B).^16^

### Secure Anonymised Information Linkage databank

We used the Secure Anonymised Information Linkage (SAIL) Databank^17–20^ that contains anonymised, routinely collected data at the individual level for all Welsh residents and all individuals who engage with the NHS in Wales.

We used the SAIL MELD-B Children and Young Adults e-cohort (SMYC), developed as part of the MELD-B project.^21^ Comprehensive details regarding the construction of this e-cohort, including data sources and demographic variables, are reported by Chiovoloni *et al*.^21^ The SMYC cohort includes individuals born between 1 January 2000 and 31 December 2022, with available residency and health data prior to the age of 18. Inclusion in the cohort required linkage to a consistent maternal record—defined as linkage to no more than one maternal identifier.^18^

For this analysis, the sample was restricted to individuals who had not had a recorded diagnosis of anxiety or depression prior to age 13. This resulted in slightly different sample sizes for the anxiety and depression analyses. Please see online supplemental material 1 for a full breakdown of how the final analytical sample was derived. Further, although the SMYC cohort includes individuals born between 1 January 2000 and 31 December 2022, the analytical sample was restricted to those who completed year 6 prior to 2020, in order to minimise potential confounding related to the COVID-19 pandemic. This restriction corresponds approximately to children born between 2000 and 2009, for whom year 6 exposure data were observed between 2010 and 2019. The outcome, defined as a first diagnosis of anxiety or depression between ages 13 and 18, was ascertained using primary care (Welsh Longitudinal General Practice (WLGP)) and hospital admissions (Patient Episode Database for Wales (PEDW)) data over the corresponding follow-up period. Depending on birth year, individuals contributed outcome data from approximately 2013 onwards, with later birth cohorts having shorter follow-up time within the available data. Finally, individuals were required to have available exposure data from the Education Wales (EDUW) datasets for year 6. EDUW covers pupils in all state-funded schools in Wales and therefore captures the overwhelming majority of the Welsh school population.

#### Outcome measurement

The outcome was a first diagnosis of either depression or anxiety between ages 13 and 18, identified either through coding in primary care records in the WLGP dataset or through hospital admissions data in the PEDW. In Wales, general practice data are coded using Read Version 2 (Read V2), while hospital admission data are coded using the International Classification of Diseases, 10th Revision (ICD-10). Accordingly, diagnostic code lists for anxiety and depression were reviewed and approved by the MELD-B clinical group.^16 21^ The complete list of Read V2 and ICD-10 codes used to diagnose anxiety and depression is provided in online supplemental material 2.

#### Exposure measurement

The exposure of interest was a domain representing childhood academic adversity, encompassing processes related to learning, educational attainment and the acquisition of knowledge within formal educational settings. All variables were derived from the EDUW dataset and measured during year 6 (ages 10–11). Exposures were analysed at the domain level—that is, as a group of conceptually related variables rather than as individual indicators. A fuller discussion of our domain-based analytical approach is provided by Stannard *et al*.^22^ To summarise, we conceptualise exposures within broader early-life domains because the variables within each domain reflect interconnected processes that operate together rather than in isolation. Considering them jointly provides a more realistic representation of the multidimensional conditions shaping children’s development, and avoids fragmenting highly related indicators into multiple statistical tests examining each component separately. Importantly, analysing domains rather than single exposures better aligns with how real-world interventions are expected to work, since early-life policies are likely to influence several aspects of the academic environment simultaneously. Treating these factors as a domain therefore reflects both their conceptual interrelatedness and the practical ways in which interventions may act across multiple pathways rather than targeting one isolated variable.

Each variable within the domain was binary and reflected a specific aspect of academic adversity at either the individual or school level. These included whether the cohort member had experienced either a fixed-term or permanent school exclusion; whether attendance rate (including both authorised and unauthorised absences) was below 90% in line with the UK government’s definition of persistent absence^23^; whether the teacher–pupil ratio (TPR) in the pupil’s class exceeded 21:1, a threshold selected as it is above the current average in England^24^; and whether a pupil failed to achieve Level 4 or above in any core Key Stage 2 subjects, English, Mathematics and Science, based on teacher assessments. This threshold aligns with the Welsh Government’s target for academic achievement at the end of year 6.^25^ Each variable was coded as 1 for adversity and 0 for no adversity. These binary indicators were then summed to create an overall domain adversity score, with higher scores indicating greater adversity. Due to the small number of participants with very high adversity scores (≥3), all individuals with a score of two or more were grouped into a single category for analysis.

#### Confounders

**Figure 1:**
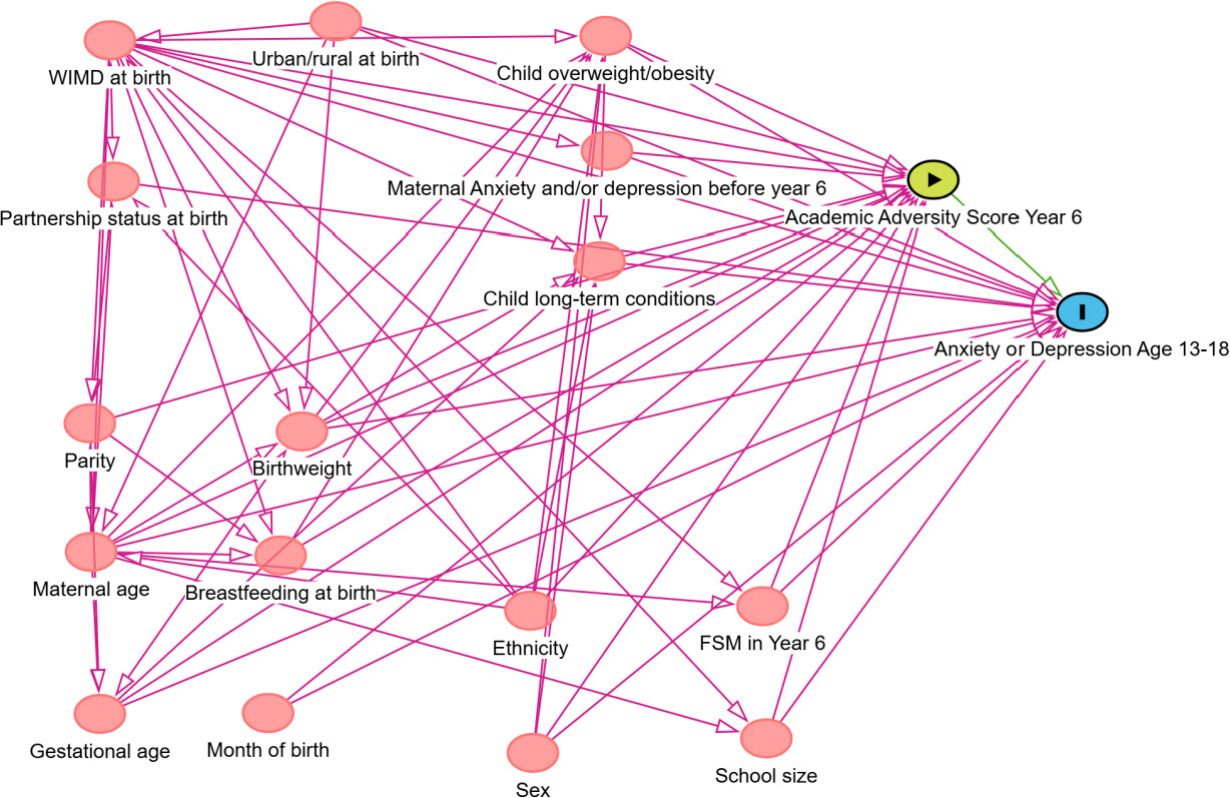
A DAG exploring the relationship between variables included in the analytical model. DAG, directed acyclic graph; FSM, free school meals; WIMD, Welsh Index of Multiple Deprivation.

We controlled for a number of covariates selected based on a priori knowledge.^26^ The selection of confounders was informed by a directed acyclic graph (DAG) using DAGitty V.3.0 software to explore the most parsimonious possible models (figure 1). The DAG confirmed the need to include all above variables in the fully adjusted model.

### Perinatal covariates

Birth weight and gestational age were categorised using standard measures^27 28^: birth weight as very low (below 1500 g), low (1500–2499 g), normal (2500–4000 g) or high (over 4000 g); gestational age as preterm (36 weeks or below), term (37–42 weeks) or post-term (42 weeks or above). Breastfeeding status at birth was recorded as yes or no.

Month of birth was derived from the week of birth using data from the Annual District Birth Extract. Maternal age at pregnancy (at first booking appointment) was recorded in years. Parity was categorised as 0, 1, 2, 3, 4 or 5+ (higher-order births were grouped due to small numbers). Partnership status at birth was derived from cohabitation data and classified as married, cohabiting, not married with a partner living at a different address or single parent.

### Other covariates

Maternal history of anxiety or depression was identified through linked primary care (WLGP) and hospital admission (PEDW) records prior to the child reaching year 6. Child overweight or obesity status (prior to reaching year 6) was obtained from the Child Measurement Programme and general practitioner records, calculated as weight/height² (kg/m²) and converted to age-adjusted and sex-adjusted z-scores using the UK 1990 growth reference. Overweight and obesity in childhood were defined as a body mass index z-score ≥91 st percentile (z ≥ +1.33).^29^ We additionally considered diagnoses of long-term conditions occurring before age 10 that could plausibly influence educational or mental health outcomes. These conditions were selected using the clinical expertise within the research team and based on a priori knowledge. The conditions were recorded in either primary care records through linked primary care (WLGP) and hospital admission (PEDW). These conditions included asthma, diabetes (type 1 and 2), cystic fibrosis, epilepsy, congenital disorders and chromosomal abnormalities, autism and attention deficit hyperactivity disorder (ADHD), blindness and low vision, deafness, eating disorders, leukaemia, lymphoma and paralysis including cerebral palsy.

Area-level deprivation at birth was measured using the Welsh Index of Multiple Deprivation, linked to the child’s birth record and categorised into quintiles (1=most deprived, 5=least deprived). Socioeconomic status during primary school was measured via the proxy of eligibility for FSM in year 6 (no/yes). Ethnicity was classified according to the Office for National Statistics five-category system: (1) Asian, Asian British or Asian Welsh; (2) Black, Black British, Black Welsh, Caribbean or African; (3) Mixed or Multiple ethnic groups; (4) White and (5) Other ethnic group. Urban or rural classification of the mother’s residence at birth was derived from birth registration data. We included one additional school-level covariate—school size.

#### Statistical analysis

Our analytical approach followed the methodology developed in Stannard *et al*,^22^ which used a domain-based adversity score, estimation of population attributable fractions (PAFs) and application of real-world intervention effect estimates to model hypothetical prevention scenarios on the reduction of multiple long-term conditions at age 46.

Multivariable logistic regression models were used to examine the association between adversity within the academic domain and a first diagnosis of anxiety or depression between ages 13 and 18. Initial models assessed unadjusted associations. Subsequent models adjusted for perinatal covariates, then other covariates were included in the models. Given well-documented sex differences in educational outcomes and the prevalence of adolescent mental health conditions,^30–34^ analyses were carried out separately for boys and girls.

From the fully adjusted logistic regression models, we calculated PAFs using the ‘punaf’ package in Stata V.17.^35^ PAFs estimate the proportion of anxiety or depression prevalence that could be prevented if someone were to reduce their adversity score. PAFs were estimated immediately following the multivariate logistic regression modelling, using the same analytical sample. The reference population for the PAFs was defined as children without a depression or anxiety diagnosis between 13 and 18, that was consistent with the reference group used in the regression analyses. This approach ensured alignment between the regression modelling and the PAF estimation, allowing for interpretable estimates of the proportion of outcome cases that could be attributable to the academic adversity domain. The multivariable logistic regression model and the PAFs modelling took account of clustering within schools, using a school-level identifier.

The full adjusted logistic regression was conducted using a complete-case approach, including only participants with no missing data on variables included in the models. We explored the use of multiple imputation to address missing data; however, this was not feasible due to computational constraints associated with the dataset size. As such, results should be interpreted with caution of potential bias introduced by missing data.

To explore the potential impact of real-world interventions, we reviewed the published evidence to identify effect estimates from existing interventions targeting factors (exclusion, attendance, attainment and TPR) within the academic adversity domain. This review informed the selection of relevant policies and programmes that could feasibly reduce domain adversity scores during childhood. Initially, we focused on effect estimates of the intervention based on UK data; however, if these were not available, we included effect estimates from other countries. Interventions were selected based on several criteria. First, we included only interventions with citable literature that reported effect estimates, which often limited our selection to well-established interventions with long-term follow-up data. Second, we focused on interventions that had been implemented in the UK, even if UK-specific evaluations were not available. Finally, we prioritised interventions that could, in principle, be rolled out universally across the UK, assuming the absence of structural or economic barriers.

We focused on two interventions that could feasibly impact academic adversity. These interventions included Sure Start—latterly known as Best Start (or Flying Start in Wales), an initiative launched in 1998 to provide area-based holistic support to families with children up until the age of 5.^36^ It achieved this through bringing together a range of services with the aim of enhancing development and life chances by providing services focusing on health, parenting support, early learning and childcare and parental employment support.^36^ The second intervention was the universal roll out of FSM during primary school.^37^ While we modelled universal FSM as an intervention, we felt this does not conflict with using FSM eligibility as a covariate to proxy socioeconomic status. The intervention scenario assumes all children receive FSM regardless of eligibility, whereas the covariate reflects baseline socioeconomic differences in the observed data. Including FSM eligibility in the adjusted models helps control for confounding by socioeconomic status.

Table 1 includes the variables within the academic adversity domain and how the effect estimates from the two interventions map onto these variables. We summarised how the intervention effect estimates were applied to each variable in order to model their impact. We were unable to find effect estimates for interventions addressing TPRs, and after discussing with the research team it was agreed there were unlikely to be specific policies that target this area.

**Table 1:** Mapping effect estimates from Sure Start and FSM to the academic ability domain

Variable within domain	Intervention	Effect estimates from evaluations of interventions	How was effect estimate modelled onto original variable of interest
Educational attainment	FSM^37^	Percentage of children reaching expected KS2 grades increased 4.2% (UK).^65^	A random sample of 4.2% of children who had not achieved expected KS2 grades were reclassified as achieved expected grades.
Attendance	FSM^37^ Sure Start^36^	1.2 fewer days missed at school over the academic year in total (UK).^38^ 0.3 fewer days missed at school over the academic year (UK).^39^	All children reduced the number of days missed from school by 1.2. All children reduced the number of days missed from school by 0.3.
Exclusions	FSM^37^ Sure Start^36^	Decrease in exclusion at Middle School of 1.9% (USA).^66^ Decrease in exclusion at age 11 of 0.5% (UK).^39^	A random sample of 1.9% of children who had an exclusion were reclassified as not having an exclusion. A random sample of 0.5% of children who had an exclusion was reclassified as not having an exclusion.
Teacher-pupil ratio	No interventions available	–	–

FSM, free school meals; KS2, key stage 2.

We modelled the absolute reduction in outcome risk under a hypothetical scenario where all cohort members were exposed to the combined effect of Sure Start and universal FSM. This required recalculating each individual’s academic adversity score using published effect estimates for both interventions. For instance, evaluations of FSM indicated that schools implementing universal FSM experienced an average reduction of 1.2 days missed school over the academic year^38^ (table 1). Sure Start participation was associated with a reduction of 0.3 days missed school in year 6^39^ (table 1). We therefore assumed that combined exposure would result in a total reduction of 1.5 days absent days and recalculated each individual’s absence rate on the basis that everyone experienced this reduction. The same approach was applied to other variables within the academic adversity domain wherever relevant effect estimates were available, such as exclusions and attainment, while TPR remained unchanged due to the absence of intervention data.

We then recalculated the academic adversity scores accounting for the effect of the intervention on reducing domain adversity scores. This allowed us to identify the total number of participants who would have reduced their domain adversity scores (eg, from 2+ to 1, from 2+ to 0 and from 1 to 0) had they been exposed to the combined effect of interventions.

Using the effect estimates from the significant PAF scenarios and applying them to the number of children (with either anxiety or depression) who reduced their domain adversity scores (as a result of being exposed to the combined effect of Sure Start and FSM), we identified the number of children who would no longer report anxiety or depression between ages 13 and 18 under these hypothetical combined prevention scenarios. In doing so, we calculated the fully adjusted absolute reduction in risk of adolescent anxiety or depression among certain domain adversity groups.

To account for within-family clustering, where multiple children are linked to the same mother, we repeated the analysis but randomly selected one sibling per family for inclusion. Siblings were identified based on having the same maternal identifier. The results are available in online supplemental materials 3 and 4 and discussed below.

#### Patient and public involvement

Patients and members of the public were not involved in the design, analysis or interpretation of this specific study. However, a Patient and Public Advisory Board established for the wider MELD-B project, of which this work forms part, provides strategic guidance on research priorities and programme direction.

## Results

### Descriptive results

**Figure 2:**
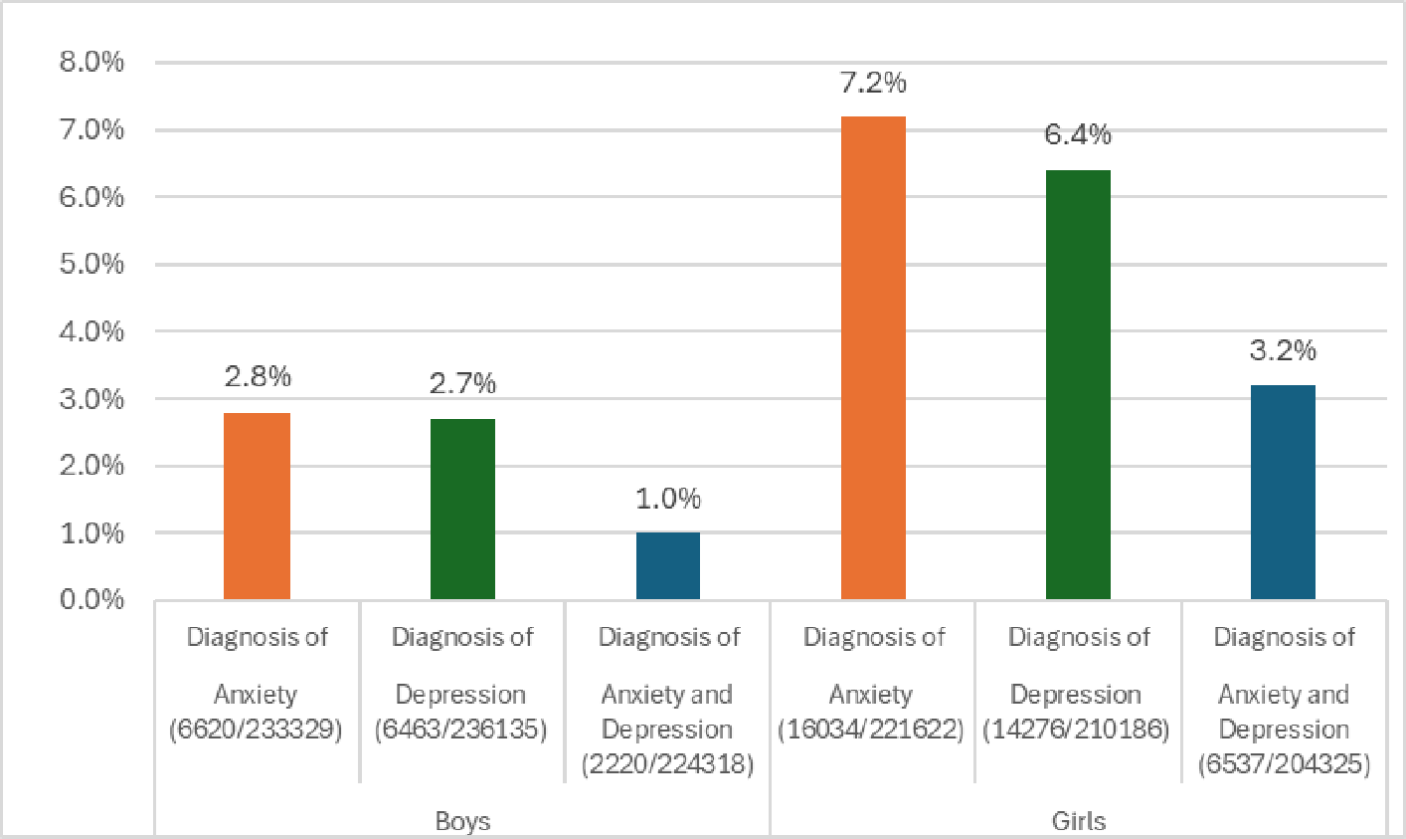
Total number and per cent of boys and girls with a first diagnosis of anxiety or depression between ages 13 and 18.

As shown in figure 2, 2.8% (6620/233 329) of boys and 7.2% (16 034/221 622) of girls had a first diagnosis of anxiety between ages 13 and 18, while 2.7% (6463/236 165) of boys and 6.4% (14 276/224 462) of girls had a first diagnosis of depression during the same period. 1.0% (2220/224 318) of boys had a first diagnosis of both anxiety and depression between ages 13 and 18, while 3.2% (6539/204 325) of girls had a first diagnosis of both conditions over the same period. Given the small number of adolescents, particularly boys, with combined anxiety and depression, we chose to conduct the rest of our modelling on anxiety and depression separately because there was insufficient statistical power to model the combined outcome.

Online supplemental material 5 includes the distribution of the reporting of a first anxiety and depression diagnosis between the ages of 13 and 18, for girls and boys and by variables included in the regression models. Descriptive statistics in online supplemental material 5 are based on all available data for each variable, leading to variation in sample sizes across rows. As missingness differs by variable, the totals reported in online supplemental material 5 do not consistently match the overall cohort sizes reported in the text. As shown, 3.0% of boys and 8.3% of girls with an academic adversity score of 0 (no adversity) had a first diagnosis of anxiety between ages 13 and 18. This prevalence rose to 3.4% for an adversity score of 1 and 4.3% for an adversity score of 2+ for boys, and to 8.7% for an adversity score of 1 and 9.4% for girls with an adversity score of 2+. Regarding depression, 3.0% of boys with an adversity score of 0, 3.3% with an adversity score of 1 and 3.9% with an adversity score of 2+ had a first diagnosis of depression between ages 13 and 18. For girls, 7.1% with an adversity score of 0, 7.9% with an adversity score of 1 and 8.4% with an adversity score of 2+ have a diagnosis of depression between ages 13 and 18.

### Multivariable logistic regression

Table 2 presents the ORs from the multivariable logistic regression of anxiety and depression according to adversity scores for boys and girls. We include three models, model 1 was unadjusted, model 2 additionally included perinatal covariates and model 3 additionally included all remaining covariates.

**Table 2:** Logistic regression testing the association between academic adversity and anxiety and depression, for boys and girls, clustered by school

		Anxiety Boys n=98 491 Girls n=93 416						Depression Boys n=100 677 Girls n=92 615					
		Model 1: Unadjusted		Model 2: +perinatal covariates*		Model 3: +other covariates†		Model 1: Unadjusted		Model 2: +perinatal covariates*		Model 3: +other covariates†	
		OR	95% CI	OR	95% CI	OR	95% CI	OR	95% CI	OR	95% CI	OR	95% CI
Boys	0	REF	REF	REF	REF	REF	REF	REF	REF	REF	REF	REF	REF
	1	**1.28**	**1.20 to 1.37**	**1.24**	**1.17 to 1.33**	**1.12**	**1.05 to 1.20**	1.22	**1.13 to 1.31**	**1.22**	**1.13 to 1.31**	1.08	0.99 to 1.17
	2+	**1.82**	**1.66 to 1.98**	**1.68**	**1.54 to 1.84**	**1.31**	**1.18 to 1.45**	**1.58**	**1.44 to 1.75**	**1.59**	**1.44 to 1.75**	**1.15**	**1.03 to 1.30**
Girls	0	REF	REF	REF	REF	REF	REF	REF	REF	REF	REF	REF	REF
	1	**1.14**	**1.09 to 1.20**	**1.12**	**1.06 to 1.17**	**1.07**	**1.01 to 1.12**	**1.22**	**1.16 to 1.28**	**1.17**	**1.11 to 1.22**	**1.09**	**1.04 to 1.15**
	2+	**1.43**	**1.32 to 1.54**	**1.36**	**1.26 to 1.47**	**1.20**	**1.10 to 1.31**	**1.51**	**1.40 to 1.63**	**1.35**	**1.24 to 1.46**	**1.12**	**1.03 to 1.23**

Estimates that are statistically significant at the 95% level shown in bold.

* Birthweight, gestational age, breastfeeding, month of birth, maternal age, parity, partnership status at birth.

† WIMD, school size, free school meal eligibility, urban or rural, ethnicity, maternal history of anxiety or depression, child overweight or obese, number of long-term conditions.

REF, reference; WIMD, Welsh Index of Multiple Deprivation.

For both boys and girls, across both outcomes and for models 1 and 2, there was a significant relationship across adversity scores, demonstrating that with an increase in academic adversity score the odds of reporting either anxiety or depression increased, compared with an adversity score of zero. This relationship was particularly strong for boys and the outcome of anxiety. In the fully adjusted models for the outcome of anxiety (table 2) and for both boys and girls, higher adversity was significantly associated with anxiety, compared with an adversity score of zero (boys 1: OR 1.12 95% CI 1.05 to 1.20; 2+: OR 1.31 95% CI 1.18 to 1.45) (girls 1: OR 1.07 95% CI 1.01 to 1.12; 2+: OR 1.20 95% CI 1.10 to 1.31). For the outcome of depression (table 2) and for girls, higher adversity was significantly associated with depression, compared with an adversity score of zero (1: OR 1.09 95% CI 1.04 to 1.15; 2+: OR 1.12 95% CI 1.03 to 1.23). For boys, an adversity score of 1 compared with 0 was not significantly associated with depression (OR 1.08 95% CI 0.99 to 1.17), but an adversity score of 2+ remained associated with depression, compared with an adversity score of 0 (OR 1.15 95% CI 1.03 to 1.30). Similar findings were observed in online supplemental materials 3 and 4 where we considered the same associations but for one sibling only as a sensitivity analysis. However, in the one sibling only model boys with an adversity score of 1 compared with 0 remained associated with depression (OR 1.14 95% CI 1.03 to 1.25) (online supplemental material 4).

### Population attributable fractions

Next we explored the PAFs in relation to the outcome of anxiety (table 3). If boys with a score of 2+ moved to a score of 1 or 0, there could be a 13.5% (95% CI 6.3% to 20.1%) and 22.1% (95% CI 14.4% to 29.1%) reduction in the reporting of anxiety in this group, respectively. If boys with an adversity score of 1 moved to a score of 0, there could be a 10.0% (95% CI 4.1% to 15.6%) reduction in the reporting of anxiety. The PAFs for girls in relation to the outcome of anxiety are shown in table 3. If girls with a score of 2+ moved to a score of 1 or 0, there could be a 9.6% (95% CI 3.9% to 14.9%) and 14.5% (95% CI 8.4% to 20.1%) reduction in the reporting of anxiety, respectively. If girls with an adversity score of 1 moved to a score of 0, there could be a 5.5% (95% CI 1.3% to 9.6%) reduction in the reporting of anxiety in this group.

**Table 3:** Adjusted population attributable fractions (PAFs) for the reduction in the reporting of anxiety or depression in adolescence with lowering academic adversity scores by the end of primary school

		Anxiety Boys n=98 491 Girls n=93 416		Depression Boys n=100 677 Girls n=92 615	
	PAF scenario	PAF	95% CI	PAF	95% CI
Boys	2+ to 1	**13.5%**	**6.3% to 20.1%**	6.2%	−2.8% to 14.4%
	2+ to 0	**22.1%**	**14.4% to 29.1%**	**12.6%**	**2.7% to 21.5%**
	1 to 0	**10.0%**	**4.1% to 15.6%**	6.9%	−0.3% to 13.6%
Girls	2+ to 1	**9.6%**	**3.9% to 14.9%**	2.2%	−5.1% to 8.9%
	2+ to 0	**14.5%**	8.4% to 20.1%	**9.3%**	**2.2% to 15.9%**
	1 to 0	**5.5%**	**1.3% to 9.6%**	**7.5%**	**3.1% to 11.6%**

Estimates that are statistically significant at the 95% level shown in bold.

The PAFs for depression are shown in table 3. Only one scenario was statistically significant for boys and two were significant for girls. If boys with an adversity score of 2+ moved to a score of 0, there could be a 12.6% (95% CI 2.7% to 21.5%) reduction in the reporting of depression among boys with an adversity score of 2+. For girls in relation to the outcome of depression (table 3), if girls with a score of 2+ moved to a score of 0, there could be a 9.3% (95% CI 2.2% to 15.9%) reduction in the reporting of depression in this group. If girls with a score of 1 moved to a score of 0, there could be a 7.5% (95% CI 3.1% to 11.6%) reduction in the reporting of depression among girls with an adversity score of 1.

### Modelling effect estimates from the interventions

This section introduces how we used the significant PAF scenarios to model the potential effect of interventions for reducing adversity scores, and subsequently a reduction in the outcome risk. We outline the number of children who would be expected to move to a lower adversity category under the intervention scenario, based on applying the published effect estimates from Sure Start and universal FSM to the variables within the academic adversity domain. We then present the estimated number of children who would no longer receive a diagnosis of anxiety or depression once these changes in adversity scores are taken into account.

#### Anxiety outcome

In table 4, we focus on three significant PAF scenarios for boys and girls: moving from 2+ to 1, from 2+ to 0 and from 1 to 0. We estimated that 92 out of 787 (11.7%) boys with an adversity score of 2+, and who reported anxiety between ages 13 and 18, would reduce their adversity score to 1. Zero out of 787 (0%) boys with an adversity score of 2+, and who reported anxiety between ages 13 and 18, would reduce their adversity score to 0, and 111 out of 1816 (6.1%) boys with an adversity score of 1, and who reported anxiety between ages 13 and 18, would reduce their adversity score to 0.

**Table 4:** Modelling the fully adjusted changes in adversity scores and subsequent absolute reduction in the reporting of anxiety, for the significant population attributable fractions (PAFs) and for boys and girls

	Significant PAF scenarios	Anxiety Boys n=98 491 Girls n=93 416			Depression Boys n=100 677 Girls n=92 615		
		Number of children reducing domain adversity score N (%)	Number of children who reduced the adversity score who would no longer report anxiety N (%)	Total number of children at risk N	Number of children reducing domain adversity score N (%)	Number of children who reduced the adversity score who would no longer report anxiety N (%)	Total number of children at risk N
Boys	2+ to 1	92 11.7	12 1.6	787	–	–	–
	2+ to 0	0 0	0 0	787	0 0	0 0	703
	1 to 0	111 6.1	11 0.6	1816	–	–	–
Girls	2+ to 1	117 10.8	11 0.5	1080	–	–	–
	2+ to 0	0 0	0 0	1080	0 0	0 0	990
	1 to 0	209 5.2	12 0.3	3986	214 5.9	16 0.4	3626

From the PAF analysis we identified that 13.5% of boys who reduced their adversity scores from 2+ to 1 would no longer report anxiety, and applying that 13.5% reduction to the 92 boys who moved from adversity score 2+ to 1, we project that 12 boys would no longer report anxiety between ages 13 and 18 (table 4). This represents a 1.6% absolute reduction in risk of the reporting of anxiety among boys with an adversity score of 2+. For the PAF scenario of 2+ to 0 we identified a potential 22.1% reduction in the reporting of anxiety, however, after modelling the interventions, no boys with an adversity score of 2+ and who had reported anxiety between ages 13 and 18 reduced their adversity score to zero, as such there was no absolute reduction in the reporting of anxiety for this group. Finally from the PAF analysis 10.0% of boys who reduced their adversity score from 1 to 0 would no longer report anxiety, and applying that 10.0% reduction to the 111 boys who moved from adversity score 1 to 0, we project that 11 boys would no longer report anxiety between ages 13 and 18 (table 4). This represents a 0.6% absolute reduction in the reporting of anxiety among boys with an adversity score of 1.

For girls, we estimated that 117 out of 1080 (10.8%) girls with an adversity score of 2+, and who reported anxiety between ages 13 and 18, would reduce their adversity score to 1. Zero out of 1080 (0%) girls with an adversity score of 2+, and who reported anxiety between ages 13 and 18, would reduce their adversity score to 0, and 209 out of 3986 (5.2%) girls with an adversity score of 1, and who reported anxiety between ages 13 and 18, would reduce their adversity score to 0.

The PAF analysis demonstrated that 9.6% of girls who reduced their adversity scores from 2+ to 1 would no longer report anxiety and applying that 9.6% reduction to the 117 girls who moved from adversity score 2+ to 1, we project that 11 girls would no longer report anxiety between ages 13 and 18 (table 4). This represents a 0.5% absolute reduction in the reporting of anxiety among girls with an adversity score of 2+. For the PAF scenario of 2+ to 0 we identified a potential 14.5% reduction in the reporting of anxiety, however after modelling the interventions no girls with an adversity score of 2+ and who had reported anxiety between ages 13 and 18 reduced their adversity score to zero, as such there was no absolute reduction in the reporting of anxiety for this group. Finally, from the PAF analysis 5.5% of girls who reduced their adversity score from 1 to 0 would no longer report anxiety and applying that 5.5% reduction to the 209 girls who moved from adversity score 1 to 0, we project that 12 girls would no longer report anxiety between ages 13 and 18 (table 4). This represents a 0.3% absolute reduction in the reporting of anxiety among girls with an adversity score of 1.

#### Depression outcome

In table 4, we focus on one significant PAF scenario for boys and two PAF scenarios for girls, including moving from 2+ to 0 (girls and boys) and from 1 to 0 (girls only). We estimated that zero out of 703 (0%) boys with an adversity score of 2+, and who reported depression between ages 13 and 18, would reduce their adversity score to 0. Therefore, despite the PAF analysis suggesting that a scenario of 2+ to 0 could lead to a potential 12.6% reduction in the reporting of depression, after modelling the interventions no boys with an adversity score of 2+ and who had reported depression between ages 13 and 18 reduced their adversity score to zero. As such there was no absolute reduction in the reporting of depression for this group.

For girls we estimated that zero out of 990 (0%) girls with an adversity score of 2+, and who reported depression between ages 13 and 18, would reduce their adversity score to 0, and 214 out of 3626 (5.9%) girls with an adversity score of 1, and who reported depression between ages 13 and 18, would reduce their adversity score to 0 (table 4). For the PAF scenario of 2+ to 0 we identified a potential 9.3% reduction in the reporting of depression, however, after modelling the interventions no girls with an adversity score of 2+ and who had reported depression between ages 13 and 18 reduced their adversity score to zero, as such there was no absolute reduction in the reporting of anxiety for this group. Finally, from the PAF analysis 7.5% of girls who reduced their adversity score from 1 to 0 would no longer report depression and applying that 7.5% reduction to the 214 girls who moved from adversity score 1 to 0, we project that 16 girls would no longer report depression between ages 13 and 18 (table 4). This represents a 0.4% absolute reduction in the reporting of anxiety among girls with an adversity score of 1.

## Discussion

Analysing data from a large representative administrative data linked dataset, we established that academic adversity (across all categories) in Year 6 was significantly associated with anxiety for both boys and girls. Higher academic adversity (across all categories) was also associated with depression among girls, while experience of highest adversity (2+) was associated with depression among boys. Fully adjusted PAFs suggested that reducing adversity could lead to a reduction in both anxiety and depression.

For anxiety, the greatest reductions were observed among boys, with an estimated decrease of 13.5% (95% CI 6.3% to 20.1%) if adversity scores were reduced from 2+ to 1, and 22.1% (95% CI 14.4% to 29.1%) if adversity scores reduced from 2+ to 0. A reduction from 1 to 0 was associated with a 10.0% (95% CI 4.1% to 15.6%) decrease in the reporting of anxiety among boys. Girls also showed reductions in the reporting of anxiety, though to a lesser extent: 9.6% (95% CI 3.9% to 14.9%) for 2+to 1, 14.5% (95% CI 8.4% to 20.1%) for 2+ to 0 and 5.5% (95% CI 1.3% to 9.6%) for 1 to 0.

For depression, only one PAF scenario was significant for boys—2+ to 0—resulting in a 12.6% reduction (95% CI 2.7% to 21.5%). Among girls, reductions of 9.3% (95% CI 2.2% to 15.9%) and 7.5% (95% CI 3.1% to 11.6%) were observed for 2+ to 0 and 1 to 0, respectively.

We quantified the potential effect of two interventions which reduce children’s experience of academic adversity. By modelling the combined effect estimates from Sure Start and universal FSM in primary school, and applying the corresponding PAFs, we demonstrated that these interventions could result in small absolute reductions in outcome risk during adolescence. Early-life interventions could lead to a modest reduction in anxiety diagnoses especially for boys (0.6%–1.6% for boys; 0.3%–0.5% for girls) and a 0.4% reduction in depression diagnoses for girls, with no measurable impact for boys.

Our paper considered the combined effect of Sure Start and universal FSM simultaneously, and we believe it to be feasible that a child could be exposed to both interventions. First, in relation to universal FSM, the UK has already outlined planned expansions to the current system.^40^ Second, Sure Start, latterly known as Best Start (or Flying Start in Wales) is a UK government programme aimed at supporting families with children under five. It provides integrated services including early education, childcare, health support and parenting advice, all delivered through local Sure Start Children’s Centres. However, it is important to reflect on the fact that the reach of Sure Start has changed substantially over time: large numbers of centres have closed since 2010,^39^ and provision is now more limited and unevenly distributed geographically, meaning that access may vary by area and deprivation.^41^ Despite this, if there were universal access to Sure Start as applied in our modelling, then because these two programmes target different but overlapping stages of the early life course, it is both feasible and potentially beneficial for families to access them concurrently or sequentially. Their combined implementation reflects a layered approach to early intervention consistent with a life course perspective.

Beyond the intervention modelling, our findings highlight clear relationships between early childhood circumstances, including childhood academic adversity, socioeconomic and family-related factors and childhood health (all considered as controls in this paper), and adolescent diagnosis of anxiety and depression. These patterns are consistent with a substantial body of life course literature evaluating the influence of perinatal factors,^42^ family structures,^43 44^ parental mental health,^45 46^ socioeconomic factors^47^ and child health^48^ on child development and mental health trajectories. These findings align with wider life course theories and evidence around the enduring impact of childhood for influencing health trajectories.^49–52^ Importantly, the combination of detailed perinatal and linked maternal records, socioeconomic measures, school-level academic indicators and adolescent mental health outcomes within a single population-level dataset is rare. The size and depth of the SAIL Databank enable us to examine these interconnected factors simultaneously and at a scale not typically achievable in cohort studies. This provides a strong foundation for understanding how childhood environments shape adolescent mental health.

Our results highlighted notable differences in how academic adversity is related to anxiety and depression. First, the control covariates had a stronger attenuating effect on the association with depression than with anxiety, suggesting that depression may be more influenced by broader environmental and familial factors we considered in our models. Second across both sexes, the relationship between academic adversity and anxiety was stronger than that with depression, with particularly pronounced effects observed among boys, indicating that school-related adversity may have a more direct impact on anxiety symptoms. This contrasts with some existing literature, which reports stronger associations between ACEs and depression than with anxiety.^53^ However, evidence from a national representative cohort of children in Australia demonstrated a stronger multivariate relationship between changes to family structure and anxiety compared with depression.^54^ While other research has found a stronger association between family socioeconomic status and adolescent anxiety compared with depression.^55^ These variations underscore that the mental health impact of childhood adversity may be exposure-specific. Our results could suggest that academic adversity has a more direct relationship on anxiety compared with depression, especially for boys. The mechanisms underlying this relationship potentially linked to academic pressure, exclusion or attendance issues warrant further investigation to inform targeted interventions, but this was beyond the scope of this paper.

Our findings contribute to a body of literature highlighting the long-term impact of academic adversity across the life course. Evidence suggests that childhood education is associated with healthcare utilisation in adulthood,^56^ and that negative school experiences are linked to increased risks of poor mental well-being, mental illness and violence victimisation later in life.^57^ The Institute for Fiscal Studies Deaton Review^58^ further underscores the broader consequences of educational inequalities, including reduced labour market prospects, lower employment rates and diminished income. Therefore, while our modelling focused on the potential impact of two interventions (Sure Start and universal FSM) on adolescent mental health, it is likely that improvements in academic adversity could have wider benefits we did not consider across multiple domains of adult life.

Sex differences in the association between academic adversity and mental health outcomes were notable. Although girls were more likely to receive a diagnosis of anxiety or depression between ages 13 and 18, the strength of association between adversity scores and these outcomes was consistently greater for boys. This suggests that boys may be more sensitive to academic adversity in terms of its impact on mental health. It could also reflect that other early life factors not captured in the present analysis, may impact girls’ mental health outcomes more than academic adversity. Some of these factors might include early pubertal timing,^59^ peer and relational stressors (eg, friendship and social support)^60^ and exposure to social and appearance-related pressures.^61^

Further, covariates, such as local area deprivation, maternal mental health and long-term health conditions, had a stronger attenuating effect on the ORs for boys than for girls, particularly in relation to anxiety. This may reflect differential pathways through which adversity influences mental health across genders. The PAF scenarios further support this interpretation. We demonstrated that reducing adversity scores hypothetically yielded a greater potential reduction in anxiety prevalence among boys than girls, indicating that targeted educational interventions may be especially impactful for boys at higher risk.

Improving childhood education is a key priority for the current Labour Government, as outlined in the Children’s well-being and Schools Bill.^62^ The Bill seeks to enhance both academic attainment and school attendance through a range of measures focused on safeguarding, support and equitable access to education. The findings presented in this study align with the objectives of the Bill, highlighting the potential benefits of improving attainment, attendance and reducing exclusions for adolescent mental health outcomes. Further, improving childhood education might not only reduce poor mental health in adolescence but long-term outcomes across the life course—economic, family health and wider health-related factors such as healthcare utilisation, given that childhood education has been found to be associated with healthcare utilisation in adulthood.^56^ This paper also supports the wider healthcare shift from sickness to prevention as outlined in the NHS 10 year plan^63^ and The Department of Health and Social Care^64^ policy paper on transforming the public health system.

This study built on a previously developed methodology.^22^ In this paper, we applied the same methodology to a substantially larger, population-level dataset to explore how modelling early-life interventions can inform prevention strategies at scale. By exploring linked health and education data, we aimed to better reflect real-world scenarios and the potential impact of interventions targeting academic adversity. This work represents a step forward in equity-focused, life-course research. We hope it encourages further methodological innovation and dialogue around how early-life prevention strategies can shape mental health trajectories and broader outcomes across the life course.

While the modelling approach offers valuable insights, it remains hypothetical and subject to uncertainty. As such, findings should be interpreted with caution. Our intention was not to present a definitive framework, but to contribute to the evolving methodological landscape that seeks to capture the complexity of early-life exposures, interventions and their health impacts. Further, as outlined in our previous work,^22^ our hypothetical modelling assumed that the effects of Sure Start and universal FSM are additive when applied in combination. However, these interventions have not been evaluated together within a single population sample, and it is possible that overlapping mechanisms could lead to diminished effects. Conversely, given that the interventions target different stages of the child life course, it is also plausible that their combined impact is underestimated.

### Strengths and limitations

This study benefitted from using the SAIL Databank, a comprehensive, individual-level, longitudinal dataset covering the entire population of Wales. This enabled robust linkage of educational exposures in Year 6 with subsequent mental health diagnoses between ages 13 and 18, identified through both primary care and hospital records. The granularity of the SAIL databank allowed for measurement of multiple indicators of adversity within the academic ability domain and facilitated adjustment for a wide range of confounders, including maternal factors. The large sample size and population-level coverage enhanced the generalisability of findings and supported the modelling of realistic, scalable prevention scenarios using real-world data.

However, there are several limitations of this study. First, we applied uniform effect estimates (from interventions) across genders, despite potential differences in intervention responsiveness between boys and girls. Due to limitations in the literature on effect estimates, gender-specific modelling was not possible. Additionally, the analysis assumed universal exposure to interventions, although some participants may have already received these. We were unable to identify this from our data, which may lead to an overestimation of population-level impact. Our modelling approach did not account for complex mechanisms such as selection bias, differential uptake or interaction effects between interventions. Further, our methods also assumed the additive effects of Sure Start and FSM, despite no existing evaluations of their combined impact.

Further, while the vast majority of individuals' data could be linked, some selection bias may have been introduced because our analyses only included participants who had complete data on all variables of interest. Additionally, the EDUW dataset includes information on pupils in state-funded learning centres, and we therefore have no educational or academic information for those who attended private schools.

Inclusion in the cohort required linkage to a consistent maternal identifier. As a result, children without stable maternal linkage, for example, those who were adopted or whose maternal record was unavailable or inconsistent, were excluded. This may have led to the under-representation of children with more complex family circumstances, who could differ in exposure to academic adversity and mental health outcomes. The use of a complete-case analysis may have introduced bias. Although the final sample remained large and likely retained adequate statistical power, the potential impact of missing data on estimates cannot be excluded.

Our adversity score included four binary indicators (exclusion, attendance, attainment and TPR), which may not fully capture the breadth of academic adversity. Other potential relevant factors such as bullying, parental involvement in schooling and teacher support and engagement were not included due to data limitations. Further, our outcome was a first diagnosis of anxiety or depression between age 13 and 18 and so we were unable to consider the severity of mental health conditions or the recurrence of a mental health condition. Further, it is worth noting that we treated anxiety and depression as binary, single episode diagnoses, although adolescent mental health is often complex and recurrent, meaning our approach necessarily simplifies this reality for analytical purposes. The outcomes were identified using lists of clinical codes from both primary care and hospital data. As a result, diagnoses of anxiety and depression were based on aggregated code lists involving multiple individual diagnoses, it was not possible to distinguish between specific types of anxiety or depressive disorders.

Our study exposure included some school-level elements, and school size was adjusted for, but there could still be clustering at the school level due to shared factors. This could lead to residual confounding from the local context, or underestimated standard errors if there is significant clustering. However, this is attenuated by the very large sample size, and the inclusion of some school-level factors.

This study used data from the Welsh SAIL Databank, and it is important to reflect on the fact that the results might not be fully generalisable to other areas of the UK or internationally due to differences in educational systems and the demographic structure of the Welsh population. In particular, the analytical population was overwhelmingly White and lacked ethnic diversity. Further, despite adjusting for a wide range of covariates, residual confounding may remain. For example, unmeasured factors such as family environments, ACEs or school-level policies could have influenced both exposure and outcome. Finally, the availability of robust, UK-based evaluation data for educational interventions was limited, necessitating the use of effect estimates from international studies, which may affect generalisability.

## Conclusions

Higher adversity scores in year 6 were significantly associated with increased odds of anxiety in adolescence for both boys and girls between ages 13 and 18. Our hypothetical modelling suggests that interventions during childhood could have a modest impact on reducing anxiety in adolescence for boys, and both anxiety and depression for girls. These findings highlight the potential value of targeting academic adversity through early-life interventions as part of broader population-level mental health prevention strategies.

## Supplementary material

10.1136/bmjopen-2026-119243 online supplemental file 1

## Data Availability

Data may be obtained from a third party and are not publicly available.
