# Supplementary material for: Does intervening to improve early academic adversity impact later mental health during adolescence? A population-based linked data modelling study in Wales
Source: BMJ Open. 2026 Aug 14;16(8):e119243. doi: 10.1136/bmjopen-2026-119243 (PMC13479487; doi:10.1136/bmjopen-2026-119243)
Supplement: Supplementary file 1 [file bmjopen-2026-119243.reviewer_comments.pdf]

PEER REVIEW HISTORY

BMJ Open publishes all reviews undertaken for accepted manuscripts. Reviewers are asked to complete a checklist review form and are provided with free text boxes to elaborate on their assessment. These free text comments are reproduced below.

ARTICLE DETAILS

Title (Provisional)

Does Intervening to Improve Early Academic Adversity Impact later Mental Health during Adolescence? A Population-Based Linked Data Modelling Study in Wales

Authors

Stannard, S; Berrington, A; Ziauddeen, N; Fraser, Simon DS; Alwan, N A

VERSION 1 - REVIEW

**Reviewer**	**1**
**Name**	**Dack, Kyle**
**Affiliation**	**University of Bristol, MRC Integrative Epidemiology Unit**
**Date**	**05-Apr-2026**
**COI**	**None**

Statistical review summary:

The paper is clear and the methods are thoroughly reported. The modelling approach and its interpretation are reasonable, and the core analytical approach I have no comments or queries about. However, the study dates and study sample size are not clearly reported, and in the results the denominators and totals vary in unexpected ways. The editors may note some causal language, but this is all confined to the modelling of hypothetical interventions and is not applied to the observational associations - in this context the wording seems fair to me.

Abstract:

1. I suggest adding the study dates and total N.

Introduction:

2. In the aims section, it reported that the paper will evaluate the exposure as a determinant of the outcomes. This is stronger language than the rest of the paper, where it is described only in terms of an association.

Methods:

3. It would help readers to state the design at the beginning of the methods (STROBE item 4), e.g. this was a retrospective longitudinal analysis of children using linked data recorded between 2000 and 2022 (these dates may be wrong - see next comment).

4. Datasets: The methods would benefit from more information about the relevant date ranges of the study, because the date range of both the exposure and outcome data is not fully reported. The exposure data ends in 2019 - when did it start? The outcome data includes individuals born between 2000-2022, but for diagnosis between the ages of 13-18 much of this date range would not be relevant. It would help to add information about the date range of the actual data used in this analysis.

Results:

5. To introduce readers to the dataset, it may be helpful to first clarify the overall sample size and its derivation: the number of relevant entries in the two datasets, how many were excluded due to lack of exposure data, and the final number. I am not really sure how much variation there is in the dates, but if it is quite wide, possibly a breakdown of the N per year of Year 6 could be helpful at understanding the temporal distribution?

6. The total N of the population changes depending on the outcome: for boys, the anxiety denominator = 233,329, depression = 236,135, both 224,318. Why is this? The only possibly relevant information in the methods was “the sample was restricted to individuals who had not had a recorded diagnosis of anxiety or depression prior to age 13” - but the phrasing sounds if there remains a single analytical sample, which excluded anyone with either condition”. If the exclusions were outcome-specific, so the depression sample excluded depression prior to 13 but not anxiety, I suggest to rephrase this section.

7. Table 2. I suggest adding the dates these statistics apply to, and if there is space, a “total” row for each column.

8. Table 2. The totals vary between variables, and do not consistently match with the sample sizes reported in the text. E.g. Boys, depression: in-text total of N = 236,165, table 2 total for the “exclusions” variable of N = 236,135, “teacher-pupil ratio” variable N = 235,307. Similarly for girls, depression in-text total N = 210,186, table 2 “teacher-pupil ratio” variable N = 223,817.

In some cases the N is higher, and sometimes lower than the in-text value.

9. Table 3. Similar to the above comments, please clarify the reasons why the N are much lower in the models compared to prior tables and text. I suspect it may be due to missing data, but if so it would be helpful to explain how this was handled in the methods.

Discussion:

10. The discussion is clear and supported by the findings. One suggested addition to the limitations: the study exposure includes some school-level elements, and school size was adjusted for, but there could still be clustering at the school level due to shared factors. This

could lead to residual confounding from the local context, or underestimated standard errors if there is significant clustering. However, this is attenuated by the very large sample size, and the inclusion of some school-level factors.

**Reviewer**	**2**
**Name**	**Hutson, Elizabeth**
**Affiliation**	**Texas Tech University Health Sciences Center, School of Nursing**
**Date**	**04-May-2026**
**COI**	**None**

This is an interesting and very well written manuscript. It is an important policy piece and could help push for these crucial programs to be funded universally.

Background

• Very well written

• While there is likely a link between academic adversity and "sure start" it is not a globally known program so should just say what it is rather than the term. The explanation of it in the discussion section was clear and concise and should be included in the introduction.

Methods

• So only children with a consistent mother were enrolled- thereby excluding children who were adopted or whose mothers died? This should be mentioned in limitations.

• Ok not to define which depressive diagnosis in the actual article, but would be helpful to know which were excluded, like adjustment disorder, MDD with psychosis, etc. But need to define what types of anxiety disorders as they can be very different. For example GAD vs Panic Disorder. Though these are included in supplementary material anxiety needs to be in the actual article. Also the supplemental tables only list out codes and even experienced clinicians don't have every code memorized for depression and anxiety. Need to write out diagnosis.

Discussion

● Page 29 line 36. True, it may mean that boys may be more sensitive to academic adversity, but it may also mean that the other factors impacting girls are greater than academic adversity. Would be helpful to note and explore some of these potential factors that were missed. Or explore why girls did not change as much.

VERSION 1 - AUTHOR RESPONSE

Reviewer: 1

Abstract:

1. I suggest adding the study dates and total N.

Thank you, these have been added to the abstract.

Introduction:

2. In the aims section, it reported that the paper will evaluate the exposure as a determinant of the outcomes. This is stronger language than the rest of the paper, where it is described only in terms of an association.

Thank you, we have reviewed this section and now describe the aim in terms of an association, we now state (p.4):

'Building on this stakeholder workshop, the aim of this paper was to explore whether a domain incorporating factors relating to academic adversity recorded in Year 6 (ages 10-11) is associated to anxiety or depression in adolescence (ages 13-18), and to model prevention scenarios within this domain on the outcome of anxiety or depression using effectiveness data of real-world interventions.'

Methods:

3. It would help readers to state the design at the beginning of the methods (STROBE item 4), e.g. this was a retrospective longitudinal analysis of children using linked data recorded between 2000 and 2022 (these dates may be wrong - see next comment).

Thank you, we have now added this to the start of the methods section (p.5).

4. Datasets: The methods would benefit from more information about the relevant date ranges of the study, because the date range of both the exposure and outcome data is not fully reported. The exposure data ends in 2019 - when did it start? The outcome data includes individuals born between 2000-2022, but for diagnosis between the ages of 13-18 much of this date range would not be relevant. It would help to add information about the date range of the actual data used in this analysis.

Thank you for this comment. We agree that the temporal coverage of the exposure and outcome data should be more clearly described.

We have now clarified the effective date ranges for both exposure and outcome data in the Methods section. Specifically:

- Although the underlying SMYC cohort includes individuals born between 1 January 2000 and 31 December 2022, our analytical sample was restricted to those who completed Year 6 prior to 2020 (to minimise potential confounding

related to the COVID-19 pandemic). This corresponds approximately to birth cohorts from 2000 to 2009, with Year 6 exposure data spanning roughly 2010 to 2019.

- The outcome (first diagnosis of anxiety or depression between ages 13 and 18) was therefore observed within the corresponding follow-up window for these cohorts, approximately from 2013 onwards, depending on birth year, with later cohorts contributing shorter follow-up.

We have now added the following in the methods section (p.5):

‘For this analysis, the sample was restricted to individuals who had not had a recorded diagnosis of anxiety or depression prior to age 13. Further, although the SMYC cohort includes individuals born between 1 January 2000 and 31 December 2022, the analytical sample was restricted to those who completed Year 6 prior to 2020, in order to minimise potential confounding related to the COVID-19 pandemic. This restriction corresponds approximately to children born between 2000 and 2009, for whom Year 6 exposure data were observed between 2010 and 2019. The outcome, defined as a first diagnosis of anxiety or depression between ages 13 and 18, was ascertained using primary care (WLGP) and hospital admissions (PEDW) data over the corresponding follow-up period. Depending on birth year, individuals contributed outcome data from approximately 2013 onwards, with later birth cohorts having shorter follow-up time within the available data. Finally, individuals were required to have available exposure data from the Education Wales (EDUW) datasets for Year 6. EDUW covers pupils in all state funded schools in Wales and therefore captures the overwhelming majority of the Welsh school population.’

Results:

5. To introduce readers to the dataset, it may be helpful to first clarify the overall sample size and its derivation: the number of relevant entries in the two datasets, how many were excluded due to lack of exposure data, and the final number. I am not really sure how much variation there is in the dates, but if it is quite wide, possibly a breakdown of the N per year of Year 6 could be helpful at understanding the temporal distribution?

Thank you, we have included the following Figure in the Supplementary materials and now refer to it on page 5. We hope this makes it clearer how the sample size was derived.

Figure X. A flow diagram summarising how the final analytical sample way derived.

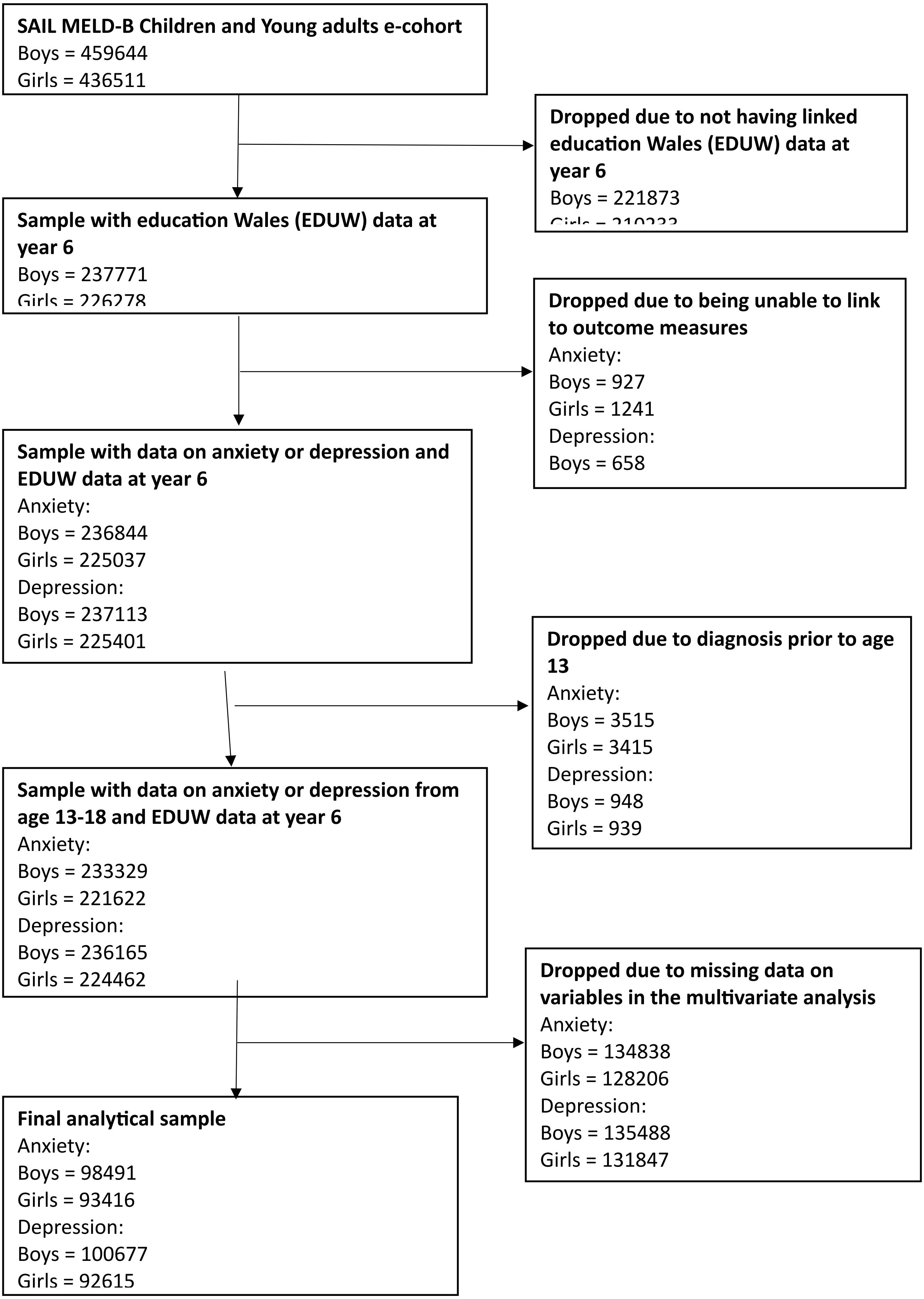

6. The total N of the population changes depending on the outcome: for boys, the anxiety denominator = 233,329, depression = 236,135, both 224,318. Why is this? The only possibly relevant information in the methods was “the sample was restricted to individuals who had not had a recorded diagnosis of anxiety or depression prior to age 13” - but the phrasing sounds if there remains a single analytical sample, which excluded anyone with either condition”. If the exclusions were outcome-specific, so the depression sample excluded depression prior to 13 but not anxiety, I suggest to rephrase this section.

Thank you, for clarity in the methods (p.5) we now state:

‘For this analysis, the sample was restricted to individuals who had not had a recorded diagnosis of anxiety or depression prior to age 13. This resulted in slightly different sample sizes for the anxiety and depression analyses. Please see Supplementary Materials 1 for a full breakdown of how the final analytical sample was derived.’

7. Table 2. I suggest adding the dates these statistics apply to, and if there is space, a “total” row for each column.

Thank you for this suggestion. We agree that clarity regarding the study time frame is important. In response to reviewer comment 4, we have expanded the Methods section to more explicitly describe the relevant exposure and outcome periods, including when the Year 6 data were recorded and the corresponding follow-up period for outcomes.

For the remaining variables, the timing of measurement is already specified in the text (e.g. at birth, during Year 6, or prior to Year 6). As this is a longitudinal cohort, adding calendar years to Table 2 would not provide meaningful additional insight. For example, perinatal variables could be recorded anywhere between 2000 and 2009. We therefore consider that the more important distinction is when in the life course the data were collected, rather than the specific calendar year.

We also considered adding a “total” row for each column. However, given the size and complexity of Table 2, and the fact that denominators vary across variables due to differences in data availability (as discussed in comment 8), we felt that including totals for each column would increase visual clutter without improving interpretability.

8. Table 2. The totals vary between variables, and do not consistently match with the sample sizes reported in the text. E.g. Boys, depression: in-text total of N = 236,165, table 2 total for the “exclusions” variable of N = 236,135, “teacher-pupil ratio” variable N = 235,307. Similarly for girls, depression in-text total N = 210,186, table 2 “teacher-pupil ratio” variable N = 223,817.In some cases the N is higher, and sometimes lower than the in-text value.

Thank you for highlighting the inconsistencies in sample sizes between the text and Table 2.

You are correct that, for each outcome, the total number reported for any variable in Table 2 should not exceed the maximum number of individuals included in that outcome-specific analytical sample (e.g. for depression: boys N = 236,135; girls N = 210,186 as reported in the text). While totals may be lower due to variable-specific missing data, they should not be higher.

The discrepancy you identified (e.g. the total for girls' depression for the teacher–pupil ratio variable exceeding N = 210,186) was due to an error in the denominator. The correct denominator for girls with depression is N = 224,462 rather than 210,186; however the percentage with depression (6.4%) was correct. This has now been amended in the manuscript.

In addition we have carefully reviewed Table 2 to ensure that:

- All variable-specific totals are consistent with the maximum eligible sample size for each outcome, and
- Any variation below these totals reflects only missing data for that specific variable.

As noted, the remaining variation in Ns across rows in Table 2 reflects differences in data availability by variable, as descriptive statistics are based on all available data rather than a complete-case sample. We clarify this on page 13:

'Descriptive statistics in Table 2 are based on all available data for each variable, leading to variation in sample sizes across rows. As missingness differs by variable, the totals reported in Table 2 do not consistently match the overall cohort sizes reported in the text.'

9. Table 3. Similar to the above comments, please clarify the reasons why the N are much lower in the models compared to prior tables and text. I suspect it may be due to missing data, but if so it would be helpful to explain how this was handled in the methods.

Thank you for this comment. The reduced N in Table 3 reflects the use of a complete-case analytical sample, whereby only participants with no missing data on variables included in the final analytical models were retained. This approach explains the lower sample size compared to prior descriptive tables.

We considered approaches to address missing data, including multiple imputation. However, due to the size and complexity of the dataset and limitations in available computational resources, this was not feasible within the scope of the study. We acknowledge that reliance on complete-case analysis may introduce bias. We have now clarified this in the Methods section and expanded the Limitations section accordingly.

Despite the reduction in sample size, the analytical sample remains large, and we believe it retains sufficient statistical power to detect meaningful associations. Previous methodological work has suggested that complete-case analysis can provide valid estimates when the proportion of missing data is modest and when models are correctly specified, although potential bias should be considered (Little & Rubin, 2019; White et al., 2011).

Additional text included in manuscript:

Methods (p.10): *'The full adjusted logistic regression were conducted using a complete-case approach, including only participants with no missing data on variables included in the models. We explored the use of multiple imputation to address missing data; however, this was not feasible due to computational constraints associated with the dataset size. As such, results should be interpreted with caution of potential bias introduced by missing data.'*

Limitations (p.33): *'The use of a complete-case may have introduced bias. Although the final sample remained large and likely retained adequate statistical power, the potential impact of missing data on estimates cannot be excluded.'*

Little RJA, Rubin DB. *Statistical Analysis with Missing Data* (3rd ed., 2019).

White IR, Horton NJ, Carpenter J, Pocock SJ. *Strategy for intention-to-treat analysis in randomised trials with missing outcome data*. BMJ. 2011.

Discussion:

10. The discussion is clear and supported by the findings. One suggested addition to the limitations: the study exposure includes some school-level elements, and school size was adjusted for, but there could still be clustering at the school level due to shared factors. This could lead to residual confounding from the local context, or underestimated standard errors if there is significant clustering. However, this is attenuated by the very large sample size, and the inclusion of some school-level factors.

Thank you for this suggestion we have added this to the limitations section (p.34).

<u>Reviewer: 2</u>
Background

1. While there is likely a link between academic adversity and "sure start" it is not a globally known program so should just say what it is rather than the term. The

explanation of it in the discussion section was clear and concise and should be included in the introduction.

Thank you, in the introduction we now state (p.4):

'We then modelled prevention scenarios within this domain on the outcome of anxiety or depression using effectiveness data of real world interventions addressing academic adversity. These interventions included free school meals and Sure Start (later known as Flying Start in Wales), an early years programme introduced in 1998 in areas based on socioeconomic deprivation to provide integrated support to families with children under five, including health services, parenting support, early learning, and childcare.'

Methods

2. So only children with a consistent mother were enrolled- thereby excluding children who were adopted or whose mothers died? This should be mentioned in limitations.

Thank you, in the limitations we now state (p.33):

'Inclusion in the cohort required linkage to a consistent maternal identifier. As a result, children without stable maternal linkage, for example those who were adopted or whose maternal record was unavailable or inconsistent, were excluded. This may have led to the under-representation of children with more complex family circumstances, who could differ in exposure to academic adversity and mental health outcomes.'

3. Ok not to define which depressive diagnosis in the actual article, but would be helpful to know which were excluded, like adjustment disorder, MDD with psychosis, etc. But need to define what types of anxiety disorders as they can be very different. For example GAD vs Panic Disorder. Though these are included in supplementary material anxiety needs to be in the actual article. Also the supplemental tables only list out codes and even experienced clinicians don't have every code memorized for depression and anxiety. Need to write out diagnosis.

Thank you, to clarify anxiety and depression outcomes were identified using routinely recorded clinical codes within linked health data, the derived clinical codes can be found following this links: https://git.soton.ac.uk/meldb/concepts. Within the coding system of the NHS and in the UK, these codes represent a broad range of diagnoses grouped under clinically defined categories (e.g. anxiety disorders, depressive disorders), rather than specific subtypes at a level that can be consistently disaggregated across large-scale routine datasets. For example, identifying 'anxiety' in a study such as this would have been done by counting any of the different diagnoses that are represented in the clinical codes under anxiety in the list if they occurred in the patient's record. As a result, it is not possible to reliably distinguish between individual conditions such as generalised anxiety disorder and panic disorder, or between different depression subtypes. This reflects an inherent limitation of routine health data

and coding systems, rather than a decision specific to this study. We have added the following to the limitations section (p.33):

'The outcomes were identified using lists of clinical codes from both primary care and hospital data. As a result, diagnoses of anxiety and depression were based on aggregated code lists involving multiple individual diagnoses, it was not possible to distinguish between specific types of anxiety or depressive disorders'.

Discussion

4. Page 29 line 36. True, it may mean that boys may be more sensitive to academic adversity, but it may also mean that the other factors impacting girls are greater than academic adversity. Would be helpful to note and explore some of these potential factors that were missed. Or explore why girls did not change as much.

Thank you, we have added the following to the discussion section (p.30):

'This suggests that boys may be more sensitive to academic adversity in terms of its impact on mental health. It could also reflect that other early life factors not captured in the present analysis, may impact girls mental health outcomes greater than academic adversity. Some of these factors might include early pubertal timing [61], peer and relational stressors (e.g. friendship and social support) [62], and exposure to social and appearance-related pressures [63].'

VERSION 2 - REVIEW

**Reviewer**	**1**
**Name**	**Dack, Kyle**
**Affiliation**	**University of Bristol, MRC Integrative Epidemiology Unit**
**Date**	**30-Jun-2026**
**COI**	

Thank you to the authors for their responses to my feedback. My primary concern previously related to the sample size and how it was derived, and this was made much clearer in the revision.

**Reviewer**	**2**
**Name**	**Hutson, Elizabeth**
**Affiliation**	**Texas Tech University Health Sciences Center, School of Nursing**
**Date**	**25-Jun-2026**

COI

Thank you for submitted a revision. I appreciate that all of the recommended revision have been completed, with the exception of you. This version is very much improved and I recommend it for publication. I do think it could be improved if you clarify if you mean depression to be MDD and anxiety to be a specific anxiety disorder. Remember the word anxiety implies the symptoms of anxiety, but there are many anxiety diagnosis including generalized anxiety disorder, separation anxiety disorder, social phobia, etc.
