# Supplementary material for: Does intervening to improve early academic adversity impact later mental health during adolescence? A population-based linked data modelling study in Wales
Source: BMJ Open. 2026 Aug 14;16(8):e119243. doi: 10.1136/bmjopen-2026-119243 (PMC13479487; doi:10.1136/bmjopen-2026-119243)
Supplement: Supplementary file 2 [file bmjopen-16-8-s001.docx]

**Supplementary Materials**

Supplimenaty materials 1. A flow diagram summarising how the final analytical sample way derived.

**Dropped due to being unable to link to outcome measures**

Anxiety:

Boys = 927

Girls = 1241

Depression:

Boys = 658

Girls = 877

**SAIL MELD-B Children and Young adults e-cohort**

Boys = 459644

Girls = 436511

**Sample with data on anxiety or depression from age 13-18 and EDUW data at year 6**

Anxiety:

Boys = 233329

Girls = 221622

Depression:

Boys = 236165

Girls = 224462

**Sample with education Wales (EDUW) data at year 6**

Boys = 237771

Girls = 226278

**Sample with data on anxiety or depression and EDUW data at year 6**

Anxiety:

Boys = 236844

Girls = 225037

Depression:

Boys = 237113

Girls = 225401

**Final analytical sample**

Anxiety:

Boys = 98491

Girls = 93416

Depression:

Boys = 100677

Girls = 92615

**Dropped due to not having linked education Wales (EDUW) data at year 6**

Boys = 221873

Girls = 210233

**Dropped due to diagnosis prior to age 13**

Anxiety:

Boys = 3515

Girls = 3415

Depression:

Boys = 948

Girls = 939

Girls = 131847

Supplementary Materials 2: READ and ICD10 codes for anxiety and depression from Chiovoloni R, Dylag JJ, Alwan NA*, et al* Cohort profile: creation of the SAIL MELD-B e-cohort (SMC) and SAIL MELD-B children and young adult e-cohort (SMYC) to investigate the lived experience of the ‘burdensomeness’ of multimorbidity *BMJ Open* 2025;15:e087946. doi: 10.1136/bmjopen-2024-087946

**Dropped due to missing data on variables in the multivariate analysis**

Anxiety:

Boys = 134838

Girls = 128206

Depression:

Boys = 135488

Girls = 131847

READ_CODE – Anxiety

1466. 146G. 1B13. 2126J 2258. 225J. 285.. 286.. 8G94. 8HHp. 8IH53 8IH57 8T23. E2... E20.. E200. E2000 E2001 E2002 E2003 E2004 E2005 E200z E201. E2010 E2011 E2012 E2013 E2014 E2015 E2016 E2017 E2018 E2019 E201A E201B E201C E201z E202. E2020 E2021 E2022 E2023 E2024 E2025 E2026 E2027 E2028 E2029 E202A E202B E202C E202D E202E E202z E203. E2030 E2031 E203z E204. E205. E206. E207. E20y. E20y0 E20y1 E20y2 E20y3 E20yz E20z. E21.. E210. E211. E2110 E2111 E2112 E2113 E211z E212. E2120 E2121 E2122 E212z E213. E214. E2140 E2141 E214z E215. E2150 E2151 E2152 E2153 E215z E216. E217. E21y. E21y0 E21y1 E21y2 E21y3 E21y4 E21y5 E21y6 E21y7 E21yz E21z. E26.. E260. E2600 E2601 E260z E261. E2610 E2611 E2612 E2613 E2614 E2615 E261z E262. E2620 E2621 E2622 E2623 E262z E263. E2630 E263z E264. E2640 E2642 E2643 E2644 E2645 E264z E265. E2650 E2651 E2652 E2653 E265z E266. E267. E26y. E26y0 E26yz E26z. E278. E2780 E2781 E2782 E278z E28.. E280. E281. E282. E283. E2830 E2831 E283z E284. E28z. E29.. E2900 E292. E2920 E2921 E2922 E2923 E2924 E2925 E292y E292z E293. E2930 E2931 E2932 E293z E294. E29y. E29y0 E29y1 E29y2 E29y3 E29y4 E29y5 E29yz E29z. E2D0. E2D0z Eu054 Eu341 Eu4.. Eu40. Eu400 Eu401 Eu402 Eu403 Eu40y Eu40z Eu41. Eu410 Eu411 Eu412 Eu413 Eu41y Eu41z Eu42. Eu420 Eu421 Eu422 Eu42y Eu42z Eu43. Eu430 Eu431 Eu432 Eu43y Eu43z Eu44. Eu440 Eu441 Eu442 Eu443 Eu444 Eu445 Eu446 Eu447 Eu44y Eu44z Eu45. Eu450 Eu451 Eu452 Eu453 Eu454 Eu455 Eu45y Eu45z Eu46. Eu460 Eu461 Eu46y Eu46z Eu515 Eu606 Eu930 Eu931 Eu932 Eu93y M240E

READ_CODE - Depression

12K8. 1465. 1B17. 1B1U. 1BT.. 212S. 2257. 62T1. 66590 6G00. 8BK0. 8CAa. 8HHq. 8HHq0 8IH52 9H90. 9H91. 9H92. 9HA0. 9Ov.. 9Ov0. 9Ov1. 9Ov2. 9Ov3. 9Ov4. 9k4.. 9k40. 9kQ.. E0013 E002. E0021 E002z E0043 E02y3 E11.. E112. E1120 E1121 E1122 E1123 E1124 E1125 E1126 E112z E113. E1130 E1131 E1132 E1133 E1134 E1135 E1136 E1137 E113z E118. E11y2 E11z2 E130. E135. E2003 E204. E290. E290z E291. E2B.. E2B0. E2B1. Eu204 Eu251 Eu32. Eu320 Eu321 Eu322 Eu323 Eu324 Eu325 Eu326 Eu327 Eu328 Eu329 Eu32A Eu32B Eu32y Eu32z Eu33. Eu330 Eu331 Eu332 Eu333 Eu334 Eu33y Eu33z Eu341 Eu3y1 Eu412 Eu530 Eu920 R007z

ICD10_CODE – Anxiety

F064 F40 F400 F401 F402 F408 F409 F41 F410 F411 F412 F413 F418 F419 F606 F930 F931 F932

ICD10_CODE – Depression

F204 F251 F32 F320 F321 F322 F323 F328 F329 F33 F330 F331 F332 F333 F338 F339 F341 F412 F920

Supplementary Materials 3. Logistic regression testing the association between education and academic adversity and anxiety, for boys and girls. Clustered by school. One sibling only.

|  |  | Model 1: Unadjusted | | Model 2: +Perinatal covariates | | Model 3: +Contextual covariates | |
| --- | --- | --- | --- | --- | --- | --- | --- |
|  |  | OR | 95% CI | OR | 95% CI | OR | 95% CI |
| Boys  n=57059 | 0 | REF | REF | REF | REF | REF | REF |
|  | 1 | **1.36** | **1.26-1.47** | **1.32** | **1.22-1.43** | **1.18** | **1.08-1.29** |
|  | 2+ | **1.90** | **1.69-2.14** | **1.75** | **1.55-1.97** | **1.33** | **1.16-1.52** |
| Girls  n=54438 | 0 | REF | REF | REF | REF | REF | REF |
|  | 1 | **1.15** | **1.09-1.22** | **1.13** | **1.06-1.20** | **1.08** | **1.02-1.15** |
|  | 2+ | **1.48** | **1.35-1.62** | **1.40** | **1.28-1.54** | **1.27** | **1.15-1.40** |

Supplementary Materials 4. Logistic regression testing the association between education and academic adversity and depression, for boys and girls. Clustered by school. One sibling only.

|  |  | Model 1: Unadjusted | | Model 2: +Perinatal covariates | | Model 3: +Contextual covariates | |
| --- | --- | --- | --- | --- | --- | --- | --- |
|  |  | OR | 95% CI | OR | 95% CI | OR | 95% CI |
| Boys  n=56728 | 0 | REF | REF | REF | REF | REF | REF |
|  | 1 | **1.27** | **1.16-1.39** | **1.21** | **1.11-1.33** | **1.14** | **1.03-1.25** |
|  | 2+ | **1.55** | **1.37-1.77** | **1.38** | **1.21-1.58** | **1.16** | **1.00-1.35** |
| Girls  n=53979 | 0 | REF | REF | REF | REF | REF | REF |
|  | 1 | **1.20** | **1.13-1.28** | **1.15** | **1.08-1.23** | **1.08** | **1.02-1.16** |
|  | 2+ | **1.58** | **1.43-1.73** | **1.40** | **1.27-1.55** | **1.21** | **1.09-1.34** |

Supplementary Materials 5. Distribution of a first anxiety and depression diagnosis, for girls and boys between the ages of 13-18 by the variables included in the regression models.

|  |  | Boys | | | | | | Girls | | | | | |
| --- | --- | --- | --- | --- | --- | --- | --- | --- | --- | --- | --- | --- | --- |
|  |  | **Anxiety** | | | **Depression** | | | **Anxiety** | | | **Depression** | | |
| Exposure |  | No | Yes | Total | No | Yes | Total | No | Yes | Total | No | Yes | Total |
|  |  | N | N |  | N | N |  | N | N |  | N | N |  |
|  |  | % | % |  | % | % |  | % | % |  | % | % |  |
| Adversity score *(including exclusions, attendance, attainment and teacher-pupil ratio)* | 0 | 94082 | 2888 | 96970 | 95258 | 2911 | 98169 | 97012 | 8751 | 105763 | 99612 | 7586 | 107198 |
|  |  | 97 | 3 |  | 97 | 3 |  | 91.7 | 8.3 |  | 92.9 | 7.1 |  |
|  | 1 | 70962 | 2477 | 73439 | 72094 | 2457 | 74551 | 58066 | 5558 | 63624 | 59516 | 5127 | 64643 |
|  |  | 96.6 | 3.4 |  | 96.7 | 3.3 |  | 91.3 | 8.7 |  | 92.1 | 7.9 |  |
|  | 2+ | 24257 | 1091 | 25348 | 24873 | 1005 | 25878 | 15516 | 1609 | 17125 | 16027 | 1474 | 17501 |
|  |  | 95.7 | 4.3 |  | 96.1 | 3.9 |  | 90.6 | 9.4 |  | 91.6 | 8.4 |  |
| Exclusions *(variable included in adversity score)* | No exclusions | 225500 | 6521 | 232021 | 228430 | 6374 | 234804 | 205449 | 16015 | 221464 | 210044 | 14252 | 224296 |
|  |  | 97.2 | 2.8 |  | 97.3 | 2.7 |  | 92.8 | 7.2 |  | 93.7 | 6.4 |  |
|  | Fixed or permanent | 1209 | 99 | 1308 | 1272 | 89 | 1361 | 139 | 19 | 158 | 142 | 24 | 166 |
|  |  | 92.4 | 7.6 |  | 93.5 | 6.5 |  | 88 | 12 |  | 85.5 | 14.5 |  |
| Attendance *(variable included in adversity score)* | 90% and over | 159305 | 4945 | 164250 | 161427 | 4975 | 166402 | 146156 | 12921 | 159077 | 149838 | 11427 | 161265 |
|  |  | 97 | 3 |  | 97 | 3 |  | 91.9 | 8.1 |  | 92.9 | 7.1 |  |
|  | 90% and under | 30536 | 1566 | 32102 | 31354 | 1439 | 32793 | 24915 | 3074 | 27989 | 25808 | 2822 | 28630 |
|  |  | 95.1 | 4.9 |  | 95.6 | 4.4 |  | 89 | 11 |  | 90.1 | 9.9 |  |
| Attainment *(variable included in adversity score)* | All KS2 results level 4 and above | 92925 | 4227 | 97152 | 92925 | 4227 | 97152 | 91369 | 12415 | 103784 | 91369 | 12415 | 103784 |
|  |  | 95.7 | 4.4 |  | 95.7 | 4.4 |  | 88 | 12 |  | 88 | 12 |  |
|  | At least one KS2 result level 3 or below | 28941 | 1798 | 30739 | 28941 | 1798 | 30739 | 14934 | 2309 | 17243 | 14934 | 2309 | 17243 |
|  |  | 94.2 | 5.9 |  | 94.2 | 5.9 |  | 86.6 | 13.4 |  | 86.6 | 13.4 |  |
| Teacher pupil ration *(variable included in adversity score)* | 20.9 or less | 186841 | 5304 | 192145 | 189144 | 5161 | 194305 | 169708 | 12662 | 182370 | 173165 | 11384 | 184549 |
|  |  | 97.2 | 2.8 |  | 97.3 | 2.7 |  | 93.1 | 6.9 |  | 93.8 | 6.2 |  |
|  | 21 or more | 39057 | 1279 | 40336 | 39733 | 1269 | 41002 | 35304 | 3308 | 386121 | 36429 | 2839 | 39268 |
|  |  | 96.8 | 3.2 |  | 96.9 | 3.1 |  | 91.4 | 8.6 |  | 92.8 | 7.2 |  |
| Birthweight | VLBW | 1512 | 104 | 1616 | 1560 | 87 | 1647 | 1361 | 182 | 1543 | 1397 | 182 | 1579 |
|  |  | 93.6 | 6.4 |  | 94.7 | 5.3 |  | 88.2 | 11.8 |  | 88.5 | 11.5 |  |
|  | LBW | 6929 | 317 | 7246 | 7063 | 345 | 7408 | 7080 | 12487 | 19567 | 7348 | 881 | 8229 |
|  |  | 95.6 | 4.4 |  | 95.3 | 4.7 |  | 87.8 | 12.4 |  | 89.3 | 10.7 |  |
|  | NBW | 96123 | 4911 | 101034 | 98338 | 4814 | 103152 | 88412 | 1276 | 89688 | 92038 | 11120 | 103158 |
|  |  | 95.1 | 4.9 |  | 95.3 | 4.7 |  | 87.6 | 12.4 |  | 89.2 | 10.8 |  |
|  | HBW | 17067 | 846 | 17913 | 17476 | 787 | 18263 | 9039 | 526 | 9565 | 9459 | 1086 | 10545 |
|  |  | 95.3 | 4.7 |  | 95.7 | 4.3 |  | 87.6 | 5.8 |  | 89.7 | 10.3 |  |
| Gestational age | Preterm | 15720 | 450 | 16170 | 15957 | 439 | 16396 | 12854 | 991 | 13845 | 13214 | 818 | 14032 |
|  |  | 97.2 | 2.8 |  | 97.3 | 2.7 |  | 92.8 | 7.2 |  | 94.2 | 5.8 |  |
|  | Term | 174520 | 5265 | 179785 | 176911 | 5133 | 182044 | 159728 | 12838 | 172566 | 163480 | 11417 | 174897 |
|  |  | 97.1 | 2.9 |  | 97.2 | 2.8 |  | 92.6 | 7.4 |  | 93.5 | 6.5 |  |
|  | Late term | 11961 | 283 | 12244 | 12092 | 266 | 12358 | 10829 | 669 | 11498 | 10986 | 635 | 11621 |
|  |  | 97.7 | 2.3 |  | 97.9 | 2.2 |  | 94.2 | 5.8 |  | 94.5 | 5.5 |  |
| Breastfeeding at birth | No | 68572 | 2645 | 71217 | 69841 | 2514 | 72355 | 61380 | 6018 | 67398 | 62939 | 5532 | 68471 |
|  |  | 96.3 | 3.7 |  | 96.5 | 3.5 |  | 91.1 | 8.9 |  | 91.9 | 8.1 |  |
|  | Yes | 79480 | 2785 | 82265 | 80796 | 2710 | 83506 | 70583 | 6992 | 77575 | 73017 | 5924 | 78941 |
|  |  | 96.6 | 3.4 |  | 96.8 | 3.3 |  | 91 | 9 |  | 92.5 | 7.5 |  |
| Month of birth | January | 19339 | 612 | 19951 | 19567 | 599 | 20166 | 17261 | 1,471 | 18732 | 17650 | 1315 | 18965 |
|  |  | 96.9 | 3.1 |  | 97 | 3 |  | 92.2 | 7.9 |  | 93.1 | 6.9 |  |
|  | February | 17074 | 512 | 17586 | 17282 | 495 | 17777 | 15566 | 1286 | 16852 | 15886 | 1176 | 17062 |
|  |  | 97.1 | 2.9 |  | 97.2 | 2.8 |  | 92.4 | 7.6 |  | 93.1 | 6.9 |  |
|  | March | 18826 | 523 | 19349 | 19071 | 568 | 19639 | 16972 | 1392 | 18364 | 17346 | 1238 | 18584 |
|  |  | 97.3 | 2.7 |  | 97.1 | 2.9 |  | 92.4 | 7.6 |  | 93.3 | 6.7 |  |
|  | April | 18303 | 529 | 18832 | 18553 | 550 | 19103 | 16232 | 1376 | 17608 | 16699 | 1148 | 17847 |
|  |  | 97.2 | 2.8 |  | 97.1 | 2.9 |  | 92.2 | 7.8 |  | 93.6 | 6.4 |  |
|  | May | 18901 | 561 | 19462 | 19142 | 532 | 19674 | 17209 | 1370 | 18579 | 17569 | 1257 | 18826 |
|  |  | 97.1 | 2.9 |  | 97.3 | 2.7 |  | 92.6 | 7.4 |  | 93.3 | 6.7 |  |
|  | June | 18621 | 539 | 19160 | 18913 | 504 | 19417 | 17057 | 1303 | 18360 | 17454 | 1171 | 18625 |
|  |  | 97.2 | 2.8 |  | 97.4 | 2.6 |  | 92.9 | 7.1 |  | 93.7 | 6.3 |  |
|  | July | 19691 | 596 | 20287 | 20010 | 584 | 20594 | 18104 | 1461 | 19565 | 18559 | 1298 | 19857 |
|  |  | 97.1 | 2.9 |  | 97.2 | 2.8 |  | 92.5 | 7.5 |  | 93.5 | 6.5 |  |
|  | August | 18954 | 568 | 19522 | 19272 | 507 | 19779 | 17220 | 1307 | 18527 | 17653 | 1140 | 18793 |
|  |  | 97.1 | 2.9 |  | 97.4 | 2.6 |  | 93 | 7.1 |  | 93.9 | 6.1 |  |
|  | September | 19730 | 505 | 20235 | 19935 | 521 | 20456 | 18132 | 1273 | 19405 | 18461 | 1155 | 19616 |
|  |  | 97.5 | 2.5 |  | 97.5 | 2.6 |  | 93.4 | 6.6 |  | 94.1 | 5.9 |  |
|  | October | 19417 | 580 | 19997 | 19680 | 552 | 20232 | 17706 | 1330 | 19036 | 18042 | 1202 | 19244 |
|  |  | 97.1 | 2.9 |  | 97.3 | 2.7 |  | 93 | 7 |  | 93.8 | 6.3 |  |
|  | November | 18003 | 527 | 18530 | 18192 | 513 | 18705 | 16335 | 1177 | 17512 | 16705 | 1017 | 17722 |
|  |  | 97.2 | 2.8 |  | 97.3 | 2.7 |  | 93.3 | 6.7 |  | 94.3 | 5.7 |  |
|  | December | 18935 | 540 | 19475 | 19161 | 509 | 19670 | 17002 | 1250 | 18252 | 17378 | 1109 | 18487 |
|  |  | 97.2 | 2.8 |  | 97.4 | 2.6 |  | 93.2 | 6.9 |  | 94 | 6 |  |
| Maternal age at pregnancy | No anxiety/depression – n (S.D) | 28.0 (6.1) | | 126946 | 28.0 (6.1) | | 129833 | 28.0 (6.1) | | 110709 | 28.1 (6.1) | | 115163 |
|  | Yes anxiety/depression – n (S.D) | 27.5 (6.3) | | 6396 | 26.9 (6.3) | | 6245 | 27.4 (6.3) | | 15445 | 27.1 (6.4) | | 13750 |
| Parity | 1 | 123410 | 3580 | 126990 | 125139 | 3578 | 128717 | 111677 | 8795 | 120472 | 114489 | 7712 | 122201 |
|  |  | 97.2 | 2.8 |  | 97.2 | 2.8 |  | 92.7 | 7.3 |  | 93.7 | 6.3 |  |
|  | 2 | 61306 | 1740 | 63046 | 62058 | 1652 | 63710 | 55259 | 4224 | 59483 | 56413 | 3744 | 60157 |
|  |  | 97.2 | 2.8 |  | 97.4 | 2.6 |  | 92.9 | 7.1 |  | 93.8 | 6.2 |  |
|  | 3 | 26345 | 791 | 27136 | 26672 | 737 | 27409 | 24048 | 1841 | 25889 | 24515 | 1647 | 26162 |
|  |  | 97.1 | 2.9 |  | 97.3 | 2.7 |  | 92.9 | 7.1 |  | 93.7 | 6.3 |  |
|  | 4 | 9269 | 279 | 9548 | 9355 | 291 | 9646 | 8792 | 699 | 9491 | 8909 | 695 | 9604 |
|  |  | 97.1 | 2.9 |  | 97 | 3 |  | 92.6 | 7.4 |  | 92.8 | 7.2 |  |
|  | 5+ | 5444 | 202 | 5646 | 5533 | 176 | 5709 | 5001 | 433 | 5434 | 5059 | 423 | 5482 |
|  |  | 96.4 | 3.6 |  | 96.9 | 3.1 |  | 92 | 8 |  | 92.3 | 7.7 |  |
| Partnership status at birth | Married/civil partnership | 55607 | 2526 | 58133 | 56943 | 2325 | 59268 | 48685 | 6232 | 54917 | 51081 | 5091 | 56172 |
|  |  | 95.6 | 4.4 |  | 96.1 | 3.9 |  | 88.7 | 11.4 |  | 90.9 | 9.1 |  |
|  | Sole registration | 9126 | 637 | 9763 | 9325 | 636 | 9961 | 8024 | 1466 | 9490 | 8177 | 1503 | 9680 |
|  |  | 93.5 | 6.5 |  | 93.6 | 6.4 |  | 84.6 | 15.5 |  | 84.5 | 15.5 |  |
|  | Cohabiting | 38087 | 1950 | 40037 | 39001 | 1915 | 40916 | 33019 | 4649 | 37668 | 34267 | 4241 | 38508 |
|  |  | 95.1 | 4.9 |  | 95.3 | 4.7 |  | 87.7 | 12.3 |  | 89 | 11 |  |
|  | Joint registration (different address) | 13084 | 754 | 13838 | 13359 | 808 | 14167 | 11176 | 1831 | 13007 | 11582 | 1713 | 13295 |
|  |  | 94.6 | 5.5 |  | 94.3 | 5.7 |  | 85.9 | 14.1 |  | 87.1 | 12.9 |  |
| WIMD Quintile | 1 (most deprived) | 44618 | 1676 | 46294 | 45280 | 1709 | 46989 | 40400 | 3956 | 44356 | 41304 | 3721 | 45025 |
|  |  | 96.4 | 3.6 |  | 96.4 | 3.6 |  | 91.1 | 8.9 |  | 91.7 | 8.3 |  |
|  | 2 | 36660 | 1276 | 37936 | 37197 | 1284 | 38481 | 32935 | 3082 | 36017 | 33812 | 2694 | 36506 |
|  |  | 96.6 | 3.4 |  | 96.7 | 3.3 |  | 91.4 | 8.6 |  | 92.6 | 7.4 |  |
|  | 3 | 33681 | 1052 | 34733 | 34238 | 1009 | 35247 | 30172 | 2671 | 32843 | 30955 | 2365 | 33320 |
|  |  | 97 | 3 |  | 97.1 | 2.9 |  | 91.9 | 8.1 |  | 92.9 | 7.1 |  |
|  | 4 | 29506 | 864 | 30370 | 29923 | 815 | 30738 | 26323 | 2045 | 28368 | 27003 | 1776 | 28779 |
|  |  | 97.2 | 2.8 |  | 97.4 | 2.7 |  | 92.8 | 7.2 |  | 93.8 | 6.2 |  |
|  | 5 (least deprived) | 28011 | 888 | 28899 | 28519 | 765 | 29284 | 25062 | 2144 | 27206 | 25961 | 1741 | 27702 |
|  |  | 96.9 | 3.1 |  | 90.4 | 2.6 |  | 92.1 | 7.9 |  | 93.7 | 6.3 |  |
| FSM eligible | No | 185088 | 4869 | 189957 | 187531 | 4683 | 192214 | 167732 | 12172 | 179904 | 171796 | 10426 | 182222 |
|  |  | 97.4 | 2.7 |  | 97.6 | 2.4 |  | 93.2 | 6.8 |  | 94.3 | 5.7 |  |
|  | Yes | 40682 | 1726 | 42408 | 41233 | 1750 | 42983 | 37017 | 3809 | 40826 | 37521 | 3816 | 41337 |
|  |  | 95.9 | 4.1 |  | 95.9 | 4.1 |  | 90.7 | 9.3 |  | 90.8 | 9.2 |  |
| Urban or rural | Urban | 103664 | 5256 | 108920 | 106144 | 5106 | 111250 | 90102 | 12752 | 102854 | 93931 | 11249 | 105180 |
|  |  | 95.2 | 4.8 |  | 95.4 | 4.6 |  | 87.6 | 12.4 |  | 89.3 | 10.7 |  |
|  | Rural | 8803 | 457 | 9260 | 9017 | 406 | 9423 | 7639 | 1055 | 8694 | 7955 | 933 | 8888 |
|  |  | 95.1 | 4.9 |  | 95.7 | 4.3 |  | 87.8 | 12.1 |  | 89.5 | 10.5 |  |
| Ethnicity | Asian | 7549 | 379 | 7928 | 7708 | 371 | 8079 | 6147 | 856 | 7003 | 6355 | 793 | 7148 |
|  |  | 95.2 | 4.8 |  | 95.4 | 4.6 |  | 87.8 | 12.2 |  | 88.9 | 11.1 |  |
|  | Black | 1146 | 21 | 1167 | 1160 | 18 | 1178 | 1066 | 51 | 1117 | 1070 | 60 | 1130 |
|  |  | 98.2 | 1.8 |  | 98.5 | 1.5 |  | 95.4 | 4.6 |  | 94.7 | 5.3 |  |
|  | Mixed | 4302 | 134 | 4436 | 3464 | 133 | 3597 | 2936 | 308 | 3244 | 2964 | 319 | 3283 |
|  |  | 96.2 | 3.8 |  | 96.3 | 3.7 |  | 90.5 | 9.5 |  | 90.3 | 9.7 |  |
|  | Other | 1227 | 26 | 1253 | 1246 | 19 | 1265 | 1076 | 77 | 1153 | 1100 | 71 | 1171 |
|  |  | 97.9 | 2.1 |  | 98.5 | 1.5 |  | 93.3 | 6.7 |  | 93.9 | 6.1 |  |
|  | White | 119436 | 6055 | 125491 | 122173 | 5920 | 128093 | 104799 | 14740 | 119539 | 109132 | 13031 | 122163 |
|  |  | 95.2 | 4.8 |  | 95.4 | 4.6 |  | 87.7 | 12.3 |  | 89.3 | 10.7 |  |
| Maternal mental health diagnosis | No | 58586 | 1847 | 60433 | 59487 | 1700 | 61187 | 52545 | 4916 | 57461 | 54179 | 4043 | 58222 |
|  |  | 96.9 | 3.1 |  | 97.2 | 2.8 |  | 91.4 | 8.6 |  | 93.1 | 6.9 |  |
|  | Yes | 63280 | 4232 | 67512 | 65200 | 4224 | 69424 | 53758 | 9830 | 63588 | 56506 | 9018 | 65524 |
|  |  | 93.7 | 6.3 |  | 93.9 | 6.1 |  | 84.5 | 15.5 |  | 86.2 | 13.8 |  |
| BMI | Normal | 88030 | 4367 | 92397 | 90062 | 4214 | 94276 | 77070 | 10639 | 87709 | 80364 | 9352 | 89716 |
|  |  | 95.3 | 4.7 |  | 95.5 | 4.5 |  | 87.9 | 12.1 |  | 89.6 | 10.4 |  |
|  | Overweight/obese | 17513 | 1027 | 18540 | 18008 | 1027 | 19035 | 14042 | 2195 | 16237 | 14566 | 2061 | 16627 |
|  |  | 94.5 | 5.5 |  | 94.6 | 5.4 |  | 86.5 | 13.5 |  | 87.6 | 12.4 |  |
|  | Not known | 15956 | 774 | 16730 | 16317 | 735 | 17052 | 14614 | 1989 | 16603 | 15190 | 1708 | 16898 |
|  |  | 95.4 | 4.6 |  | 95.7 | 4.3 |  | 88 | 12 |  | 89.9 | 10.1 |  |
| School size | No anxiety/depression – n (S.D) | 208.8 (142.4) | | 131913 | 208.8 (142.5) | | 134882 | 209.0 (143.1) | | 115364 | 209.3 (143.5) | | 119916 |
|  | Yes anxiety/depression – n (S.D) | 204.1 (135.9) | | 6552 | 204.7 (130.9) | | 6402 | 208.6 (140.1) | | 15936 | 206.8 (137.6) | | 14211 |
| Any LTC prior to 10 | No | 50319 | 2047 | 52366 | 50266 | 1859 | 52125 | 48068 | 5741 | 53809 | 48618 | 4817 | 53435 |
|  |  | 96.1 | 3.9 |  | 96.4 | 3.6 |  | 89.3 | 10.7 |  | 91 | 9 |  |
|  | Yes | 43503 | 2622 | 46125 | 43378 | 2370 | 45748 | 33955 | 5652 | 39607 | 34552 | 4628 | 39180 |
|  |  | 94.3 | 5.7 |  | 94.8 | 5.2 |  | 85.7 | 14.3 |  | 88.2 | 11.8 |  |
